# Location Is Everything: Evaluating the Effects of Terrestrial and Marine Resource Subsidies on an Estuarine Bivalve

**DOI:** 10.1371/journal.pone.0125167

**Published:** 2015-05-18

**Authors:** Joel M. S. Harding, Michelle R. Segal, John D. Reynolds

**Affiliations:** 1 Earth to Ocean Research Group, Department of Biological Sciences, Simon Fraser University, Burnaby, BC, Canada; 2 The Hakai Institute, Heriot Bay, BC, Canada; University of Auckland, NEW ZEALAND

## Abstract

Estuaries are amongst the world’s most productive ecosystems, lying at the intersection between terrestrial and marine environments. They receive substantial inputs from adjacent landscapes but the importance of resource subsidies is not well understood. Here, we test hypotheses for the effects of both terrestrial- and salmon-derived resource subsidies on the diet (inferred from stable isotopes of muscle tissue), size and percent nitrogen of the soft-shell clam (*Mya arenaria*), a sedentary estuarine consumer. We examine how these relationships shift across natural gradients among 14 estuaries that vary in upstream watershed size and salmon density on the central coast of British Columbia, Canada. We also test how assimilation and response to subsidies vary at smaller spatial scales within estuaries. The depletion and enrichment of stable isotope ratios in soft-shell clam muscle tissue correlated with increasing upstream watershed size and salmon density, respectively. The effects of terrestrial- and salmon-derived subsidies were also strongest at locations near stream outlets. When we controlled for age of individual clams, there were larger individuals with higher percent nitrogen content in estuaries below larger watersheds, though this effect was limited to the depositional zones below river mouths. Pink salmon exhibited a stronger effect on isotope ratios of clams than chum salmon, which could reflect increased habitat overlap as spawning pink salmon concentrate in lower stream reaches, closer to intertidal clam beds. However, there were smaller clams in estuaries that had higher upstream pink salmon densities, possibly due to differences in habitat requirements. Our study highlights the importance of upstream resource subsidies to this bivalve species, but that individual responses to subsidies can vary at smaller scales within estuaries.

## Introduction

Cross-ecosystem resource linkages can structure and stabilize recipient communities [1,2]. Resource linkages, or subsidies, can be driven by abiotic mechanisms [3,4], and biological processes [5,6]. The effects of subsidies can also vary among ecosystems [7,8], individuals [9,10], and with the timing, quality or quantity of resource inputs [10,11]. Interface and hydrologically-linked landscapes such as estuaries have a particularly high potential to benefit from subsidies as upstream resources are conveyed downstream, providing nutrient inputs to these low-lying recipient ecosystems [12–14]. Estuaries are at the intersection of terrestrial, freshwater and marine ecosystems and provide a conduit for the movement of resources among landscapes [1,15]. They are productive, depositional and open ecosystems [16,17], capable of receiving substantial resource inputs from external sources [18–20]. However, the importance of resource subsidies in estuarine ecology remains less clear.

Locally-derived resources within estuaries have been thought to be of primary importance [21,22]. However, more recent work has shown that externally-derived resources can form a major component of available estuarine resources [23–25]. The magnitude of resource subsidy influx can also scale with the size of upstream ecosystems and stream flow [19,26]. Many of the previously mentioned studies have centered largely on the use of stable isotopes to investigate subsidy effects. Although they are a powerful tool in ecology, enabling us to trace resource pathways and relative contributions of potential energy sources [27,28], they are limited beyond confirmation of resource assimilation [11]. Consequently less is known about the biological importance of subsidies in estuaries.

Terrestrial-derived resources are often thought to be of lower quality than estuarine or marine sources [21]. Although they can elevate organic content in estuaries [19,29,30], few studies have attempted to detect productivity responses from them [31–33] and even fewer have tested the degree to which responses can vary across landscapes [26]. Although estuaries are one of the most productive habitats globally [16], this likely varies even at regional scales as a result of the complex interactions with surrounding landscapes.

Around the Northern Pacific Rim, many estuaries also receive pulsed ‘counter-flow’ inputs of enriched marine-derived material from the annual migration of Pacific salmon (*Oncorhynchus* spp.). Because Pacific salmon die in streams after spawning, they can function as one-way nutrient vectors, acquiring the majority of their body mass at sea [34], then transporting this mass back to natal streams. Their carcasses, which are relatively rich in nitrogen and phosphorus, are scattered throughout streams and riparian habitats by predators, scavengers and water flow. Due to the higher trophic level of salmon, and contrasts in biochemistry between marine and terrestrial systems, salmon nutrient subsidies can be differentiated from terrestrial sources using stable isotope ratios of nitrogen and carbon (δ^15^N and δ^13^C); where salmon-derived material is enriched and terrestrial-derived material is depleted in heavy isotopes [26,27,35]. The net effects of Pacific salmon in coastal ecosystems can vary [26,36], ranging from nutrient subsidies through excretion and deposition of eggs and carcasses [35,37,38], to benthic disturbance and nutrient export from juvenile salmon emigration and adults digging and defending nests [39–41]. In addition to streams and forests, estuaries also receive substantial amounts of salmon-derived nutrients from upstream watersheds [42–44]. Although dissolved nutrient concentrations increase in estuaries during salmon spawning [44,45] and many estuarine organisms are known to consume carcasses [46], few studies have investigated the importance of salmon subsidies in these communities [26,44,47].

Sedentary consumers such as bivalves not only provide an opportunity to investigate the importance of terrestrial- and salmon-derived resource subsidies in estuarine food webs, but also how these relationships might change spatially within, and across, landscapes. Bivalves integrate isotopes over time and can thus reveal resource contributions in relation to proximity of resource inputs [22,48,49]. Suspension feeders such as the soft-shell clam (*Mya arenaria*) are widespread in estuaries of the Pacific Northwest and have recently been shown to assimilate terrestrial-derived resources [50]. Similar to the river continuum concept of Vannote et al. [51], we hypothesize that estuarine organisms, such as the soft-shell clam, are influenced by resources derived from both upstream and marine landscapes, and that the importance of these resources will vary spatially with landscape traits. We further hypothesize that responses of sedentary consumers can vary based on their proximity to resource subsidies and local habitat conditions.

Here, we test hypotheses on how terrestrial and salmon resource subsidies, in addition to individual traits, explain the diet (inferred from stable isotopes of nitrogen and carbon), size, and percent nitrogen of soft-shell clams (Tables 1 and 2). We test for the effects of these covariates across 14 estuaries that span natural gradients in watershed size, spawning salmon density and other attributes (Table 3). Prior to our main analyses we tested metrics of chum (*Oncorhynchus keta*), pink (*O*. *gorbuscha*) and total (chum and pink combined) salmon density for their ability to explain isotope ratios of soft-shell clam muscle tissue. We hypothesized pink salmon may have a disproportionately large effect on bivalves because they spawn further downstream than chum salmon, including upper reaches of estuaries, and thus closer to bivalve habitats.

**Table 1:** Hypotheses for soft-shell clam stable isotope ratios (δ^15^N and δ^13^C).

Variable	Mechanism	Metric	Level	Response	Reference
Salmon density	Salmon tissues are enriched in stable isotopes.	2006–2007 mean pink salmon biomass density (kg m^-2^)	Site	Positive	[37,38,47,49,96]
Watershed size	Terrestrial-derived material is depleted in stable isotopes.	Watershed size principal component axis 1 (PC1)	Site	Negative	[19,48–50,97]
Size	Larger individuals grow more slowly and have slower tissue turnover rates, which reflect dietary sources over longer time periods.	Mass (g)	Individual	Positive	[98]
Age	Older individuals have more time to accumulate stable isotope ratios from enriched dietary sources.	Age (years)	Individual	Positive	[85,99]
Intertidal height	Individuals higher in intertidal will have lower isotopic discrimination as a result of more limited feeding opportunities.	Height above datum depth (m)	Within-site	Positive	[100,101]
Temperature	Energy requirements for maintenance and growth increase with temperature, reducing isotopic discrimination.	Maximum weekly average temperature (MWAT°C)	Site	Positive	[101]
Clam bed zone	1) Moving outward from upper to lower zones (increasing distance from stream outlet) will reduce the effect of both salmon density and watershed size. 2) Moving outward from upper to lower zones will also correspond with increased dominance of marine resources and enrich isotopes.	Upper, middle and lower clam bed locations.	Within-site	1) Negative (in interaction with salmon and watershed size. 2) Positive as main effect.	[44,47–49]
Location	1) The effect of salmon and watershed size will increase going from control to below stream locations. 2) Clams below streams will experience increased influx of terrestrial resources, and therefore have depleted isotopes, compared to control locations.	Below stream and control sites.	Within-site	1) Positive (in interaction with salmon and watershed size). 2) Negative as main effect	[44,47–49]

**Table 2:** Hypotheses for soft-shell clam size and tissue %N.

Variable	Mechanism	Metric	Level	Response	Reference
Salmon density	Salmon tissues are higher quality than other sources, resulting in larger individuals and higher N content in tissues.	2006–2007 mean pink salmon biomass density (kg m^-2^)	Site	Positive	[35,42,73,76,89,102]
Watershed size	Terrestrial-derived material can enhance organic content in estuaries, which could increase clam size and %N in tissues. It is also thought to be of lower quality and may displace higher-quality estuarine resources, reducing size and %N.	Watershed size principal component axis 1 (PC1)	Site	Positive/no effect/ negative	[19,21,29,49,75,76]
Size (for %N only)	Larger individuals grow more slowly and have slower tissue turnover rates, which will reflect higher-quality dietary sources over longer time periods.	Mass (g)	Individual	Positive	[73,89]
Age	1) Size: Older individuals are larger. 2) %N: Younger individuals grow faster, resulting in higher percentages of nitrogen in their tissues.	Age (years)	Individual	1) Positive (for size) 2) Negative (for %N)	[73,89,103]
Intertidal height	Individuals located higher in intertidal will have limited feeding opportunities This should result in smaller sizes and reduced N content (energy stores) in tissues.	Height above datum depth (m)	Within-site	Negative	[100,101]
Temperature	Energy requirements for maintenance and growth increase with temperature, reducing opportunity for growth and energy stores.	Maximum weekly average temperature (MWAT°C)	Site	Negative	[101]
Clam bed zone	1) Moving outward from upper to lower zones (increasing distance from stream outlet) will reduce the effect of both salmon density and watershed size on mass and %N. 2) Moving outward from upper to lower zones will correspond with an increase in size and %N as marine resource availability increases.	Upper, middle and lower clam bed locations.	Within-site	1) Negative (in interaction with salmon and watershed size). 2) Positive as main effect	[104,105]
Location	1) The effect of salmon and watershed size will increase going from control to below stream locations. 2) Clams below streams will be smaller and have less %N compared to control locations as a result of shifting from marine- to terrestrial-dominated resources. However, reduced habitat quality in control sites may offset this effect.	Below stream and control sites.	Within-site	1) Positive (in interaction with salmon and watershed size). 2) Positive/ Negative as main effect	[104,105]

**Table 3:** Site-level covariates used to create watershed size PC1 (catchment area, bankfull, depth and bank height), percent alder, pink salmon density, temperature and distances between clam sampling locations.

Site	Catchment area (km^2^)	Mean bankfull width (m)	Mean depth (m)	Mean bank height (m)	Watershed Size PC1	Percent riparian alder	Mean 2006–07 pink salmon adult biomass density (kg/m^2^)	Maximum weekly average temperature (°C)	Distance between clam bed zones below streams (m)	Distance between zones in control locations (m)	Lateral distance between control and below stream locations (m)	Latitude	Longitude
Ada	10.1	11.1	0.12	0.34	-0.91	3.26	0.047	16.00	1 location	NA	NA	52.0553	-128.0507
Bullock Main	3.3	10.9	0.08	0.26	-2.18	3.31	0.078	19.93	35	70	140	52.4029	-128.0785
Clatse	32.1	22.8	0.16	0.30	0.53	26.08	0.264	23.38	102.5	205	280	52.3455	-127.8476
Codville	2.4	3.3	0.18	0.24	-2.50	0.00	0.004	18.92	15.5	NA	NA	52.0790	-127.8633
Fannie Left	35.0	12.8	0.16	0.39	0.39	1.74	0.090	18.57	57.5	115	60	52.0426	-128.0668
Fell Creek	7.0	10.9	0.19	0.41	-0.38	1.16	0.229	21.41	1 location	1 location	74	52.4336	-128.0790
Hooknose	18.4	16.9	0.18	0.46	0.67	3.08	0.057	18.88	56.5	113	155	52.1249	-127.8370
Kunsoot Main	5.7	13.1	0.04	0.22	-2.20	0.00	0.259	17.16	35	NA	NA	52.1569	-128.0435
Mosquito Bay	5.2	9.7	0.11	0.21	-1.84	6.33	0.081	20.48	10	1 location	70	52.3968	-128.1660
Neekas	17.6	17.7	0.16	0.40	0.33	13.35	0.413	22.84	85	170	70	52.4509	-128.1569
Quartcha	40.9	34.1	0.24	0.55	3.28	17.95	0.010	18.77	82	1 location	375	52.5155	-127.8421
Rainbow	13.7	15.1	0.23	0.47	0.77	20.34	0.001	24.86	35	NA	NA	52.4512	-127.7280
Roscoe Main	33.6	23.5	0.28	0.56	2.70	54.77	0.000	24.63	62.5	125	240	52.4696	-127.7448
Sagar	36.6	15.5	0.25	0.43	1.34	0.21	0.013	18.13	1 location	NA	NA	52.0959	-127.8388

### Study Area

**Fig 1:**
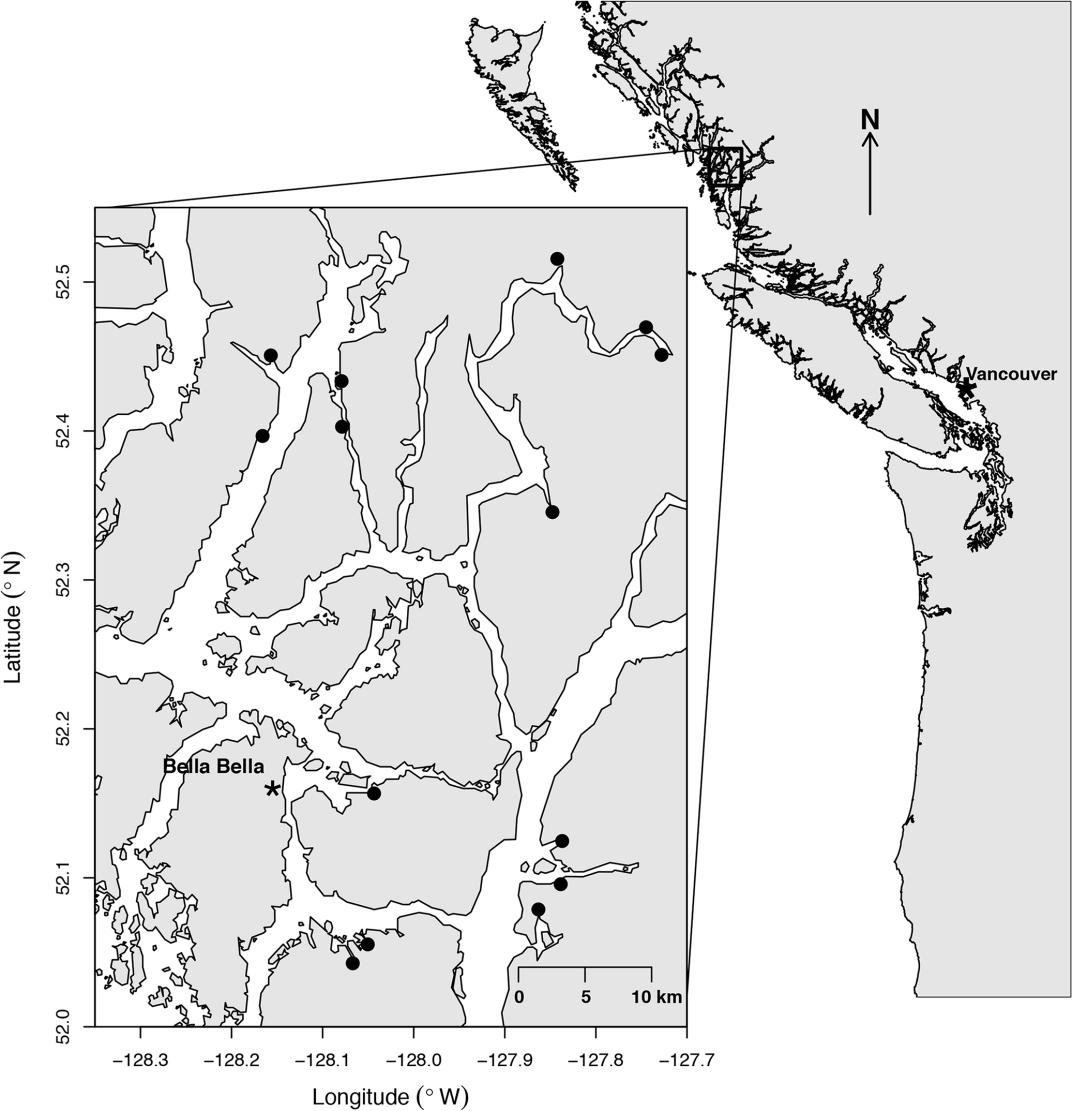
Study area in the vicinity of Bella Bella, on British Columbia’s central coast.

We studied estuaries within 45 km of Bella Bella (52°9’N, 128°8’W) on the central coast of British Columbia, Canada (Fig 1). This region lies within the Coastal Western Hemlock biogeoclimatic zone and receives some of the highest levels of precipitation on the continent [52]. Although selective logging occurred in many areas during the first half of the 20^th^ century, this region remains relatively intact due to its remoteness, restricted access and strengthening First Nations governance and conservation coalitions [53]. This remote region provides access to a wide range of relatively pristine watersheds that are ideal systems to test for the effects of terrestrial and salmon resource subsidies in estuaries.

We sampled 14 small- to medium-sized estuaries, which hosted soft-shell clam populations and varied in upstream catchment area, stream channel size, estuary area, upstream salmon spawning density, and red alder (*Alnus rubra*) dominance (Table 3). All streams were dominated by chum (*O*. *keta*) and pink (*O*. *gorbuscha*) salmon, which accounted for 90–100% of total adult salmon spawners, with much smaller numbers of coho (*O*. *kisutch*) and a limited presence of sockeye (*O*. *nerka*) and Chinook salmon (*O*. *tshawytscha*). Salmon spawn in streams throughout BC’s central and north coasts, which can produce over half of the wild salmon stocks in this region, and account for over 30% of total populations within BC and the Yukon [54].

## Methods

### Ethics Statement

Salmon counts and measurements were conducted to meet the requirements of the Canadian Council on Animal Care (approval number 1031B-11). Bivalve sampling and associated protocols at all locations were approved by Fisheries and Oceans Canada (Scientific Licence numbers XHAB 318 2008; XMCFR 11 2009).

### Sampling

**Fig 2:**
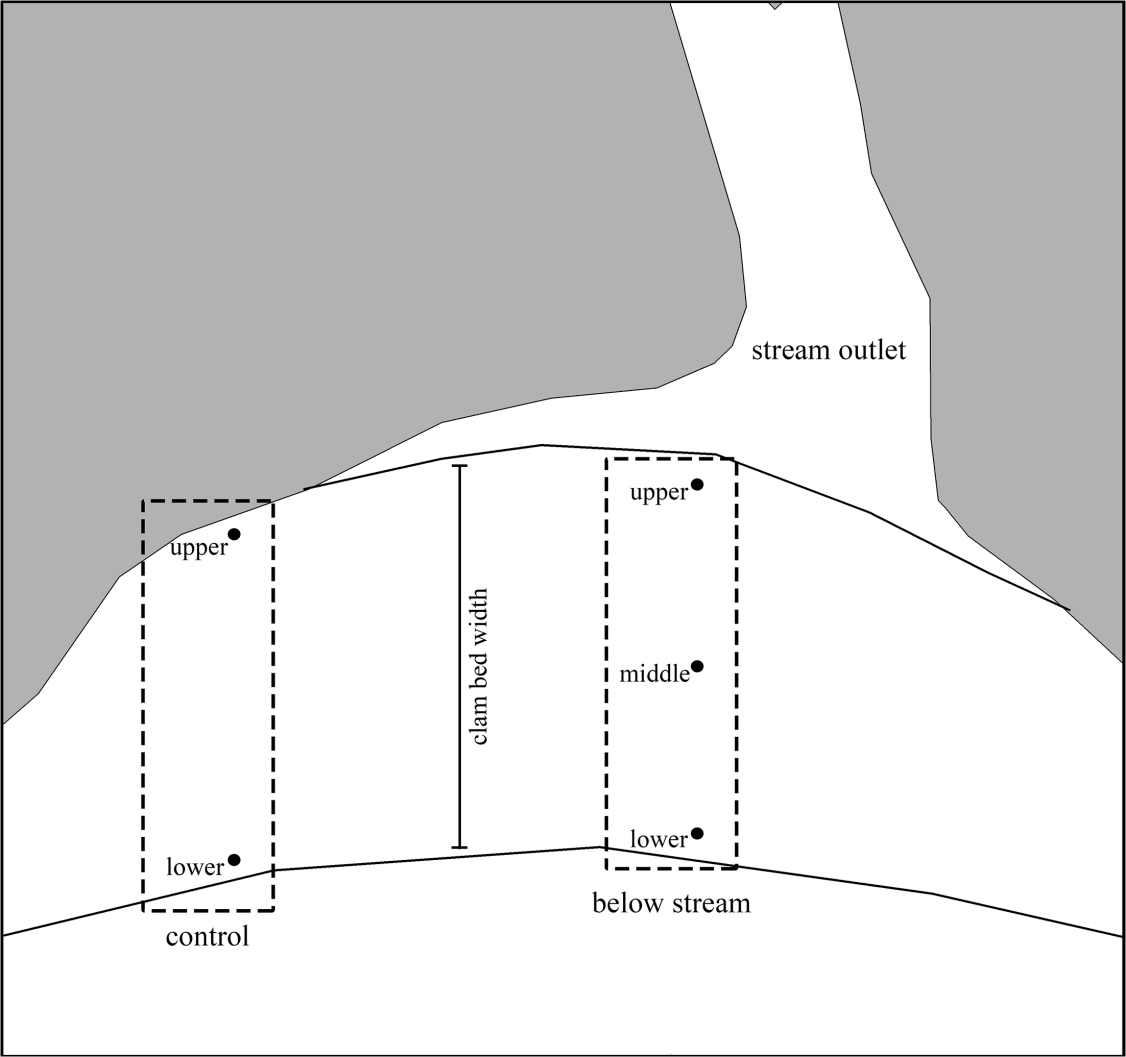
Sampling design. Upper, middle and lower clam bed zones were sampled below streams in 2008 and 2009, upper and lower zones were sampled in control locations in 2009.

We collected soft-shell clams during the summers of 2008 and 2009 prior to salmon spawning. Samples were collected during tide heights less than, or equal to 1m above chart datum depth (0 m tidal height). Depths of sample locations ranged between 0.47 and 2.1 m above chart datum. At each site three systematic locations were sampled representing upper, middle, and lower zones of the clam bed spanning the vertical width of the clam bed (Fig 2). These three zones were sampled directly below stream outlets and adjacent to the main channel within each estuary tidal flat. At each location, 5 soft-shell clams were sampled haphazardly by digging to a depth of 30 cm at each sample location and piling the sediment on the beach surface. The excavated sediment was then searched where we retained the first 5 clams encountered. This method helped reduce depth biases in sampling smaller clams in surficial sediments. Additional holes were excavated adjacent to the original if fewer than 5 clams were present. For each clam collected, we immediately recorded shell length, width, depth and wet weight. Clams were then wrapped in aluminum foil and frozen at -20°C in sealed containers until processing. Sampling time and height above water were recorded for each location to enable depth corrections to chart datum. Height above water was measured by viewing a metre stick, located at the water’s edge, through a clinometer from each sample location. The height above water was equal to the height on the metre stick, at zero degrees, minus the height of the observer’s viewpoint. In 2009, additional within-site control locations were sampled laterally down shore from steam outlets and outside the depositional deltas of each estuary (Table 3). These control locations were located in 9 of our 14 study sites and limited to the upper and lower clam bed zones (Fig 2). Age data were collected by sectioning shell chondrophores (encased in Loctite Hysol epoxy) using a Buehler Isomet Low-speed saw with diamond wafering blades. Chondrophore sections were mounted on glass slides and polished sequentially with 30, 9 and 3 micron lapping film. Sections were aged by counting annual growth lines following the methods of MacDonald and Thomas [55] using light manipulations and a digital camera mounted to a dissecting microscope.

### Watershed Data

Stream and riparian canopy (% alder) data were collected during the summer of 2007 as part of an extensive survey in the region. Temperature was measured continuously using waterproofed temperature loggers (iButtons DS1922L) anchored to rebar below chart datum depth (0m tide) and set to record every 2 hours spanning the study period. Stream measurements occurred at 12 randomly selected transects along a study reach equal to 30 times the mean bankfull width of each stream [56]. Alder basal area was estimated by measuring the diameter at breast height of all trees greater than 5 cm in diameter within six belt transects that extended perpendicular from each stream and were 35 m long by 10 m wide [35]. Percent alder was calculated for each site as:

$$\%A=\frac{B_{alder}}{B_{total}}\times 100$$

where %*A* is the percent alder for each site, *B*_*alder*_ is total basal area of all alder measured in a given site and *B*_*total*_ is the total basal area of all tree species measured in that site. Watershed catchment areas were estimated using the Government of British Columbia’s mapping website *iMap*BC [57].

Principal components analysis (PCA) was used to generate a composite variable to describe watershed size to approximate the magnitude of stream discharge and amount of terrestrial-resource influx into estuaries. Component variables included total catchment area (km^2^), mean stream bankfull width (width of the stream channel at its highest point before flooding), mean stream depth, and mean stream bank height (maximum stream depth before flooding). Pearson correlation coefficients of component variables ranged between 0.7 and 0.9. The first principal component axis (PC1) described 80% of component variable variances and variables all loaded positively on this axis ranging between 0.48 and 0.52. The PC1 axis values reflect both the capacity of streams to transport nutrient subsidies into estuaries (stream channel measurements) and the amount of terrestrial-derived nutrient sources upstream (catchment area).

### Salmon Population Data

The federal Department of Fisheries and Oceans, the Heiltsuk Integrated Resource Management Department, and Simon Fraser University cooperatively conducted all salmon enumeration and spawning channel measurements. We considered upstream salmon biomass density estimates between 2006 and 2009 as potential proxies for salmon carcass availability in estuaries downstream [26]. We determined this from data limitations (data collection began in 2006 and we did not want to consider years beyond 2009). Salmon biomass density indices were calculated for chum salmon, pink salmon and chum and pink salmon combined, for year combinations 2006–2007, 2006–2008 and 2006–2009 for each site:

$$SBD_{ij}=\frac{\sum \left(N_{ij}\times W_{j}\right)}{A}$$

where *SBD*_*ij*_ = average kg of salmon biomass per m^2^ of spawning area per stream for year combination *i* and species *j*, *N*_*ij*_ = the mean number of returning adult salmon for year combination *i* and species *j*, *W*_*j*_ = average salmon mass for each species *j*, and *A* = the estimate of spawning area (m^2^) within each stream. Spawning area was estimated by multiplying the mean bankfull width by the total spawning channel length for each stream. We accounted for variation in salmon body mass among populations by measuring the weight of 5 dead adult salmon of each sex for each species in a subset of study streams covering our study area. These average salmon masses were applied to the remaining study sites sharing island groups, channels or mainland inlets. We limited our analyses to chum and pink salmon because these species account for 90–100% of total adult salmon in our study region.

We conducted an initial exploratory analysis to identify the best salmon density metric that explained stable isotope ratios of clam foot muscle tissue. We constructed univariate linear models with chum, pink or total (chum and pink) salmon density for each selected year combination explaining δ^15^N or δ^13^C. We competed these models using Akaike Information Criterion corrected for small sample sizes (AICc) that selects for the most parsimonious model given the data. We log transformed all salmon density metrics in all analyses to reduce the leverage of high salmon density values on slope estimates.

### Stable Isotope Analysis

Foot muscle tissue samples for isotope analysis were removed from thawed samples and placed in a drying oven at 58°C for up to 96 hours. Each sample was homogenized into a fine powder using a heavy duty Wig-L-Bug grinder (Pike Technologies Ltd). Sample weights ranging between 0.8–1.2μg were packaged in standard pressed tin capsules (3.5 x 5 mm) and sent to the UC Davis Stable Isotope Facility for analysis of nitrogen and carbon abundance using a PDZ Europa ANCA-GSL elemental analyzer interfaced to a PDZ Europa 20–20 isotope ratio mass spectrometer (Sercon Ltd., Cheshire, UK). Stable isotopes are expressed as the difference between the sample and a known standard, or δ, in parts per thousand (‰):

$$\delta^{15}N\;or\;\delta^{13}C=\left(\frac{R_{sample}}{R_{standard}}-1\right)\times \;1000$$

where *R* is the ratio of the heavy isotope to the light isotope (^15^N/^14^N or ^13^C/^12^C).

Standards for nitrogen and carbon analysis are derived from N_2_ in air and Pee-Dee Belemnite (PDB) limestone, respectively.

Percent nitrogen of soft-shell clam muscle tissue was calculated as:

$$\%N=\frac{N}{T}\times 100$$

where *N* is the mass of nitrogen in the sample and *T* is the total mass of the sample.

### Statistical Analyses

Bivalve mass was chosen as the most ecologically meaningful metric representing an individual’s size [58]. We used the open source statistical software R for all analyses [59]. Variance inflation factors (VIF) of all covariates were less than 2.2 and thus indicated low multicolinearity [60]. Pearson correlation coefficients between individual covariates were 0.6 or less and not of great concern [61]. The only exception was % alder, which had a VIF of 4.3 and Pearson correlation coefficients of approximately 0.8 with both watershed size and temperature. Due to this high collinearity % alder was removed from all analyses.

For all analyses (isotopes, mass and %N) we used linear mixed-effects modeling to account for the hierarchical structure of the data [62]. This method allowed regression intercepts to vary by site (site as random intercept), accounted for potential correlation of individuals from the same site between sample years (correlation structure of site within year for all analyses), and accounted for heterogeneity in the residual variance structure [60]. Correlation and variance structures were established from residuals of the global models, or models including all variables considered, and AICc selection of the most parsimonious structures with the global model using restricted maximum likelihood (REML) estimation [60]. Variance structures on datum depth and control/below stream covariates improved the likelihood of the global models and satisfied the assumptions of residual normality and equal variance for mass and %N analyses respectively [60]. No variance structures were required for isotope analyses as the assumptions of equal variance were already met. We include a pseudo-R^2^ value for the averaged model from each analysis. This is the R^2^ value for a linear model between the fitted values of the averaged model and the observed data. We conducted an additional analysis on an approximation of clam growth that we calculated as individual clam mass divided by age, or the average mass acquired per year. Results were very similar to our analysis of clam mass so we chose to not include it to avoid redundancy.

We wanted to test how the effects of salmon and watershed subsidies could vary by distance from stream outlets (upper, middle and lower clam bed zones) and by location (control vs. below stream). We therefore constructed our models to include the following interactions in all analyses; salmon and zone, salmon and location, watershed size and zone and watershed size and location. We competed models of all combinations of covariates in addition to the specified interactions because we did not have any *a priori* reason to exclude any models from the analyses [63]. For each analysis we conducted two model competitions, first using centered covariates (subtracting the mean) and again using scaled covariates (centering and dividing by 2 standard deviations). All covariates were centered to avoid inaccuracies in slope estimates for main effects as they can vary considerably depending on the presence of interaction terms [64]. We also analyzed models with scaled covariates to enable direct comparison of effect sizes amongst variables [65,66]. In all analyses, *k*-1 binary dummy variables were created for the three-level zone factor (upper, middle, lower) and 2-level location factor (control, below stream), where *k* is the number of levels in a factor following the methods of Schielzeth [64]. In standardized models, dummy variables were not divided by 2 standard deviations as slope estimates from binary variables already relate to 2 standard deviations (comparisons of 0 and 1) [66]. Model competition using AICc revealed that top model weights in all analyses were less than 0.22. We accounted for this model uncertainty using a multi-model approach [67]. Candidate models used in multi-model inference were limited to the subset of models with a ΔAICc less than 4 [63] and estimates for each covariate and interaction term were averaged across candidate model sets using the natural average method. Intercepts, slopes, scaled coefficients and standard errors for the combined effects of salmon and watershed size at each zone and location level were calculated from averaged model outputs. The equations used to calculate these combined effects from interactions are presented in the supplemental information.

## Results

### Sampling

A total of 154 and 243 soft-shell clams were sampled in 2008 and 2009 respectively, from 14 sites each year. Clam mass ranged between 1.3–116.9 g and 3.2–126.6 g in 2008 and 2009, respectively. In 2009, control samples were collected from 9 of the 14 sites (Fig 2). Bedrock and small estuary sizes prevented control sampling from the remaining sites. Table 3 provides a summary of site-level covariates and distances between sampling locations.

### Salmon Metric Pre-selection

The 2006–07 mean pink salmon density explained the most variation in both δ^15^N and δ^13^C of soft-shell muscle tissue with model weights exceeding 0.8. This salmon metric was used in all subsequent analyses. Model rankings are presented in S1 Table.

### Clam Isotopes

#### δ15N

**Fig 3:**
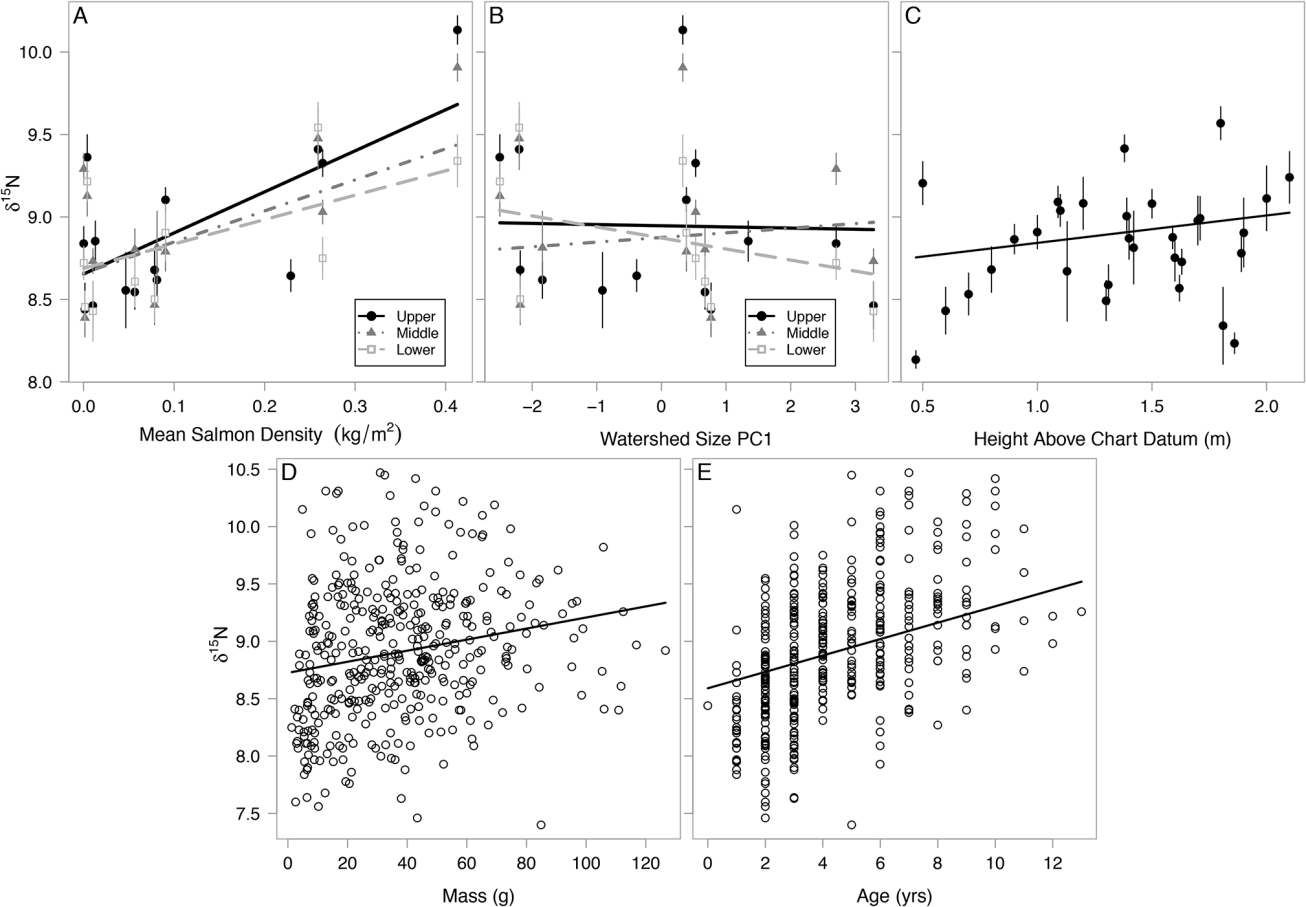
Correlates of soft-shell clam muscle tissue δ^15^N. (A) Pink salmon density at each clam bed zone, (B) Watershed size PC1 at each clam bed zone, (C) Height above datum depth, (D) Clam mass, and (E) Clam age. Each data point in panels A-C represents mean values with standard error bars. Data points in panels D-E represent individual clams. All trend lines represent relationships using intercept and coefficients from multi-model output; thus they represent the relationships for the x-axis variable that accounts for the effects of other variables, rather than fitting the univariate data shown in each graph.

**Fig 4:**
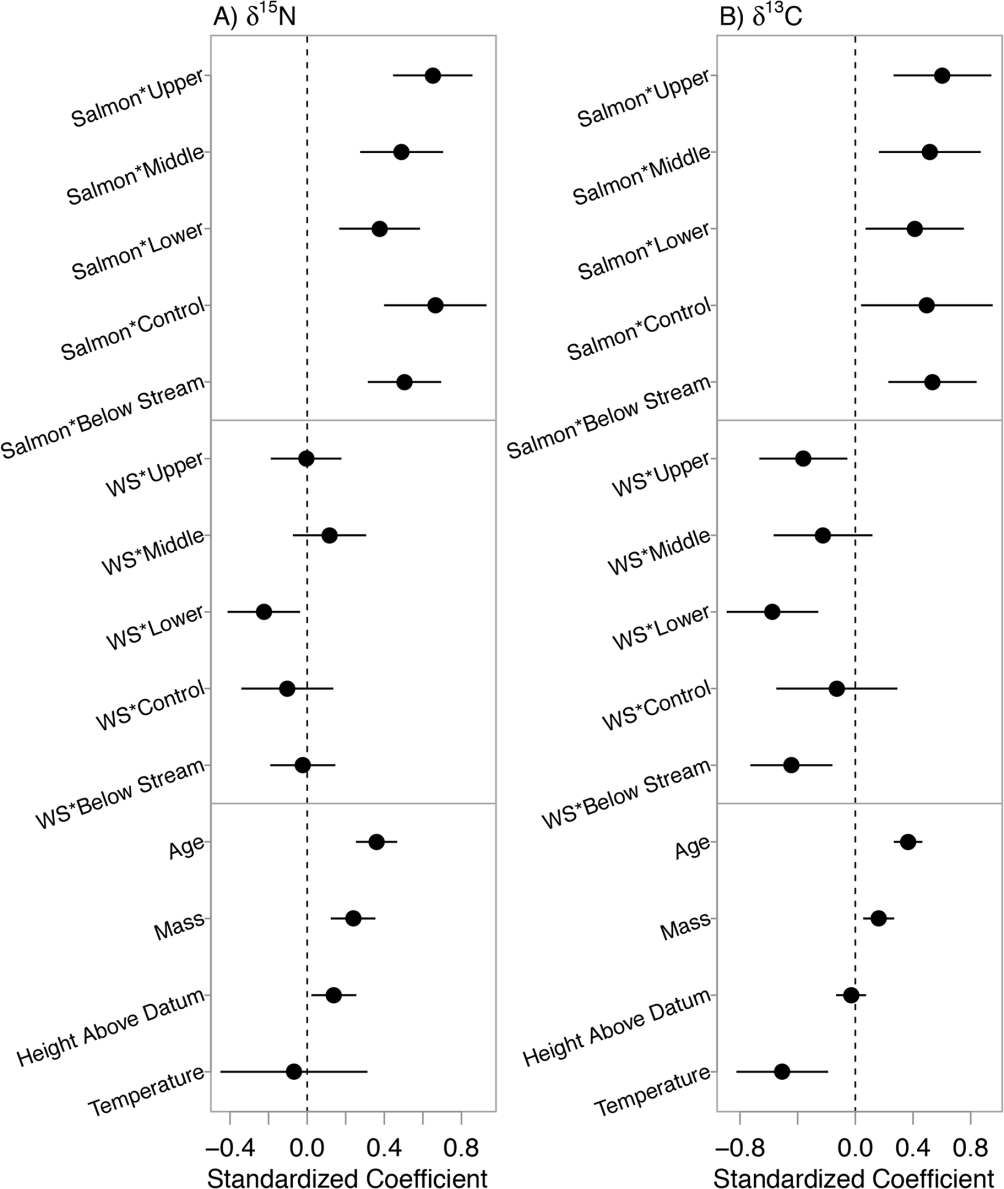
Standardized coefficients (mean = 0, standard deviation = 2) with 95% confidence intervals for covariates considered in the (A) δ^15^N candidate model set and (B) δ^13^C candidate model set. Salmon = 2006–07 mean pink salmon density; WS = watershed size PC1. Coefficient values indicate the change, on average, in δ^**15**^N or δ^**13**^C as the associated covariates increase by 2 standard deviations.

The pseudo- R^2^ of the averaged model was 0.66. Following our predictions, clams in estuaries with higher upstream salmon densities had enriched δ^15^N. In addition, the effect of salmon decreased going from upper to lower clam beds (Fig 3A). Clams below streams with large watersheds were more depleted in δ^15^N, but this was only detected in the lower zones (Fig 3B). Clams that were higher on shore (higher above chart datum), and those that were larger and older had enriched δ^15^N, following our predictions (Fig 3C–3E). Temperature did not describe δ^15^N. The standardized effects of salmon were positive at all zone and location levels with confidence intervals well above 0 (Fig 4A). The effects of watershed size were more variable, with confidence intervals crossing 0 with the exception of lower clam beds (Fig 4A). Age, mass and height above chart datum were all positive and highly certain while the effects of temperature were small and high a higher degree of uncertainty (Fig 4A). The averaged δ^15^N model and candidate set are presented in S2 and S3 Tables.

#### δ^13^C

**Fig 5:**
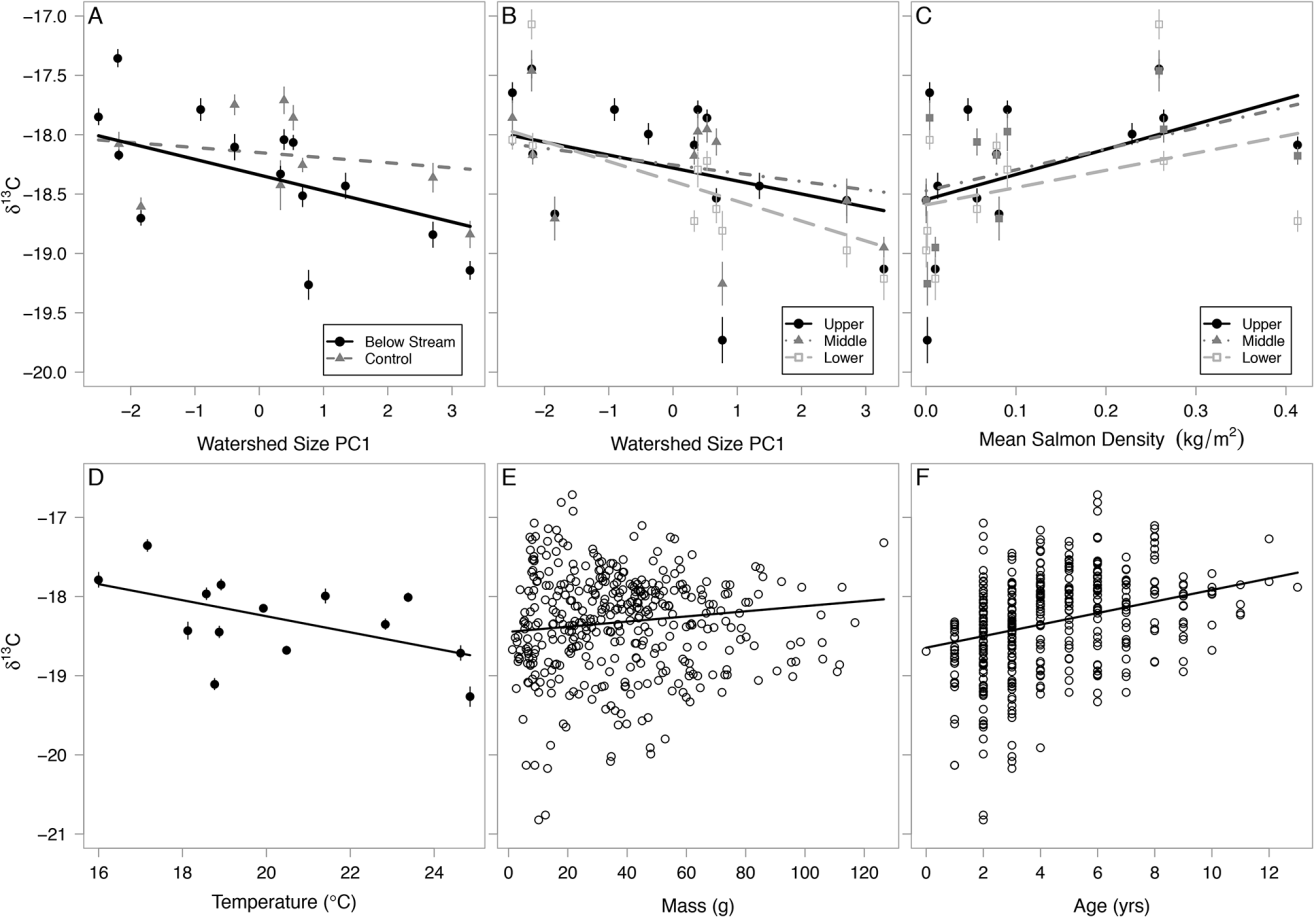
Correlates of soft-shell clam muscle tissue δ^13^C. (A) Watershed size PC1 at below stream vs. control locations, (B) Watershed size PC1 at each clam bed zone, (C) Pink salmon density at each clam bed zone, (D) Temperature (maximum weekly average temperature), (E) Clam mass, and (F) Clam age. Each data point in panels A-D represents mean values with standard error bars. Data points in panels E-F represent individual clams. All trend lines represent relationships using intercept and coefficients from multi-model output; thus they represent the relationships for the x-axis variable that accounts for the effects of other variables, rather than fitting the univariate data shown in each graph.

The pseudo- R^2^ of the averaged model was 0.23. Clams below large watersheds were more depleted in δ^13^C and, as we predicted, this depletion was strongest in the locations below streams (Fig 5A) but also in the lower zones compared to the upper and middle zones (Fig 5B), which did not support our predictions. Clams were enriched in δ^13^C below streams with higher pink salmon densities and this effect was stronger in the upper and middle zones (Fig 5C). Contrary to our predictions, warmer estuaries had clams with more depleted δ^13^C (Fig 5D). Larger and older clams were enriched in δ^13^C (Fig 5E and 5F) but height above chart datum did not have any effect (Fig 4B). Similar to δ^15^N, the standardized effects of salmon on δ^13^C were positive at all zone and location levels with confidence intervals above 0 (Fig 4B). The standardized effects of watershed size were strongest in the upper and lower zones, and below stream locations but less certain in middle zones and control locations (Fig 4B). The effects of age, mass and temperature had a high degree of certainty around coefficient estimates (Fig 4B). The averaged δ^13^C model and candidate set are presented in S4 and S5 Tables.

### Size

**Fig 6:**
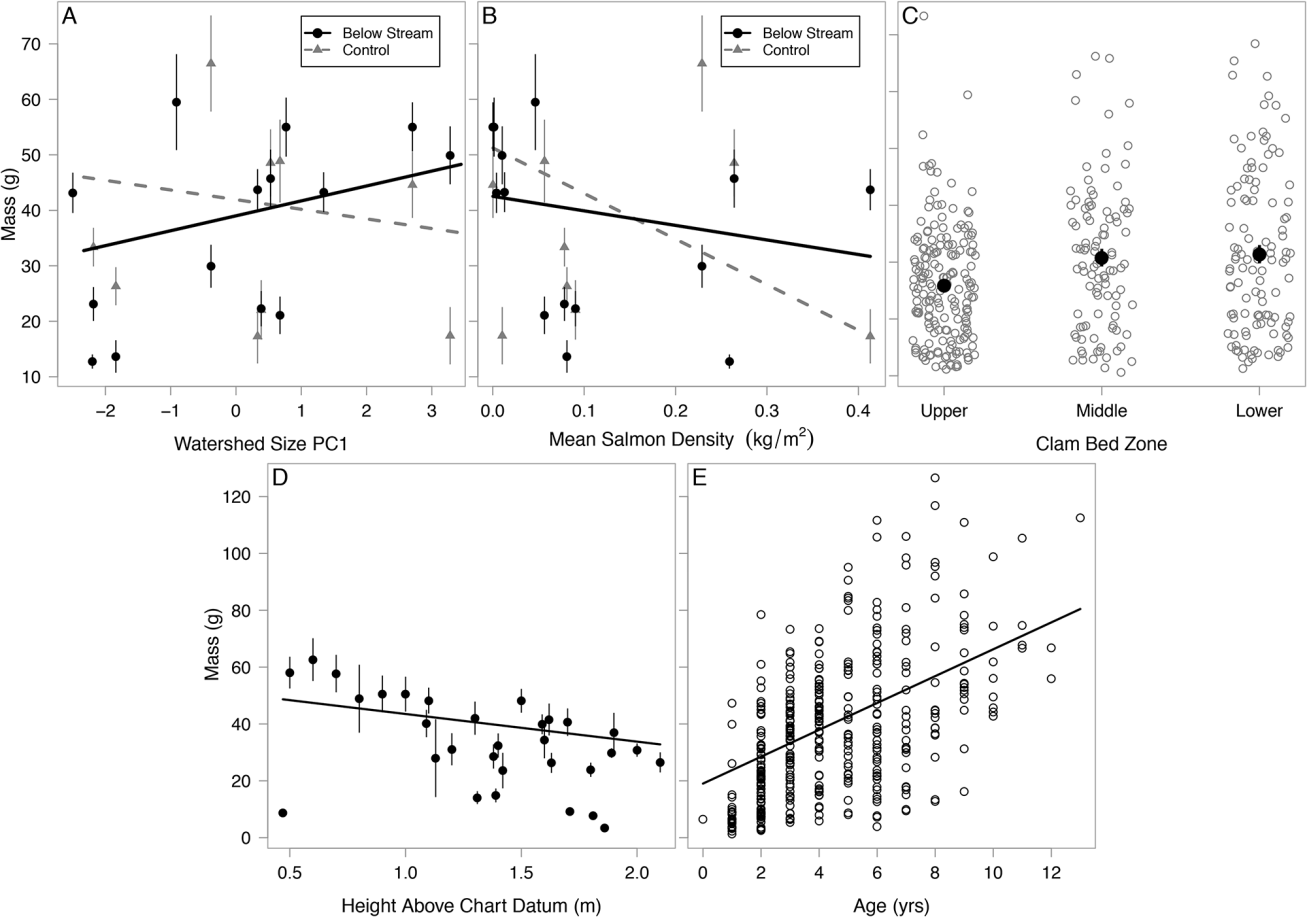
Correlates of soft-shell clam size. (A) Watershed size PC1 at below stream vs. control locations, (B) Pink salmon density at below stream vs. control locations, (C) Clam bed zone, (D) Height above chart datum, and (E) Clam age. Each data point in panels A, B and D represents mean values with standard error bars. Data points in panels C and E represent individual clams. Solid circles in panel C indicate mean mass for each zone with standard error bars. A jitter function was used in panel C for better visualization. All trend lines represent relationships using intercept and coefficients from multi-model output; thus they represent the relationships for the x-axis variable that accounts for the effects of other variables, rather than fitting the univariate data shown in each graph.

**Fig 7:**
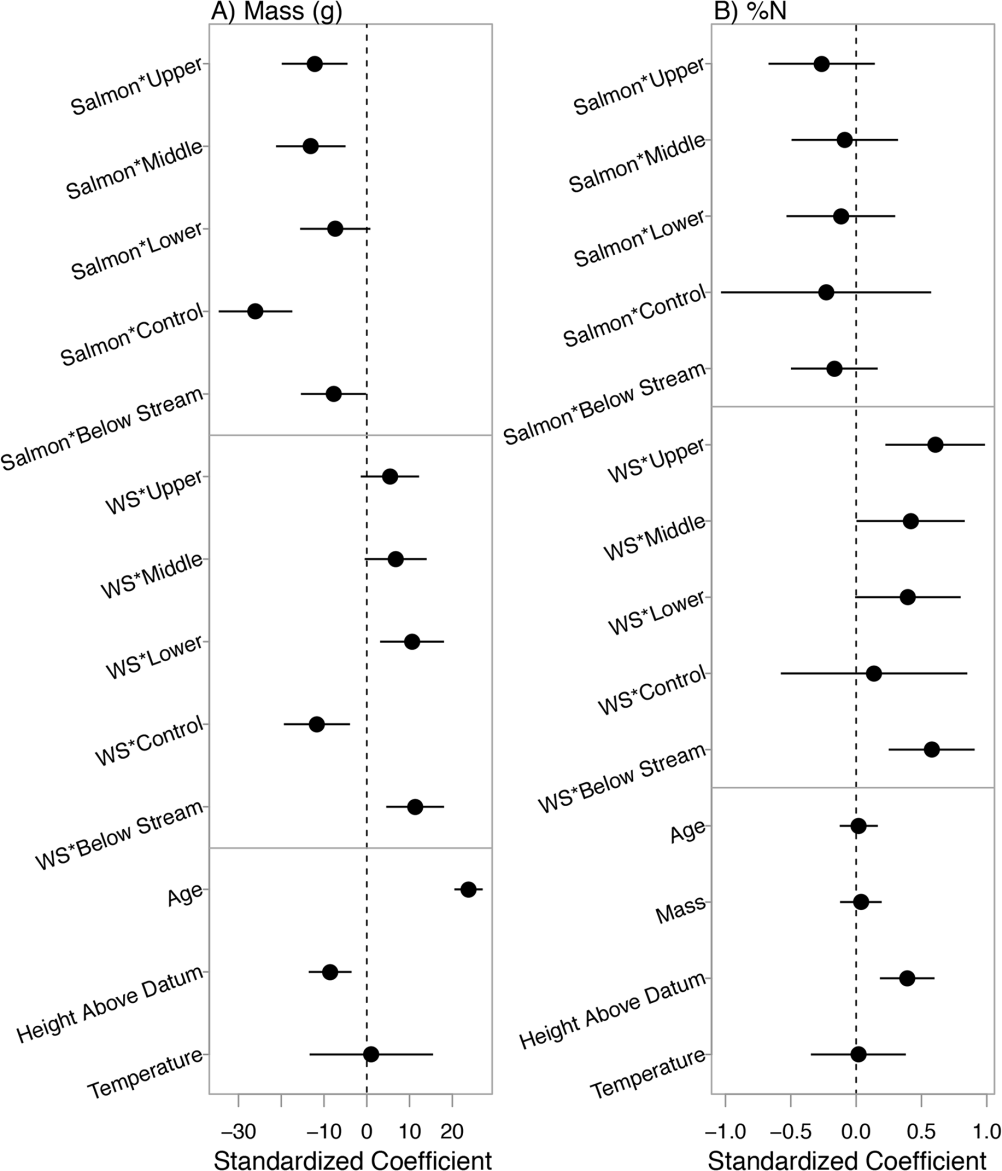
Standardized coefficients (mean = 0, standard deviation = 2) with 95% confidence intervals for all covariates considered in the (A) Soft-shell clam size candidate model set and (B) Soft-shell clam %N candidate model set. Salmon = 2006–07 mean pink salmon density; WS = watershed size PC1. Coefficient values indicate the change, on average, in clam size or %N as the associated covariates increase by 2 standard deviations.

The pseudo- R^2^ of the averaged model was 0.62. There were larger clams below larger watersheds but this effect was restricted to below stream locations (Fig 6A). Watershed size showed the opposite, and negative, correlation with size in control locations (Figs 6A and 7A). The positive correlation between clam size and watershed size was strongest in lower clam beds, and below stream locations where confidence intervals did not cross 0 (Fig 7A). The effect of location (below stream vs. control) was not an important descriptor of clam mass on its own. Surprisingly, salmon correlated negatively with clam size, opposite to our predictions, though the correlation with salmon in the below stream locations was less negative (Figs 6B and 7A). This negative relationship was observed at all zone and location levels, where most of the confidence intervals did not cross 0 (Fig 7A). Clams were also slightly smaller in the upper, compared to middle and lower clam beds (Fig 6C), and clams that were higher above chart datum and younger were smaller (Fig 6D and 6E). The effects of age and height above chart datum were positive and negative respectively, with a higher degrees of certainty, while the effects of temperature were negligible. The averaged clam size model and candidate set are presented in S6 and S7 Tables.

### Percent N

**Fig 8:**
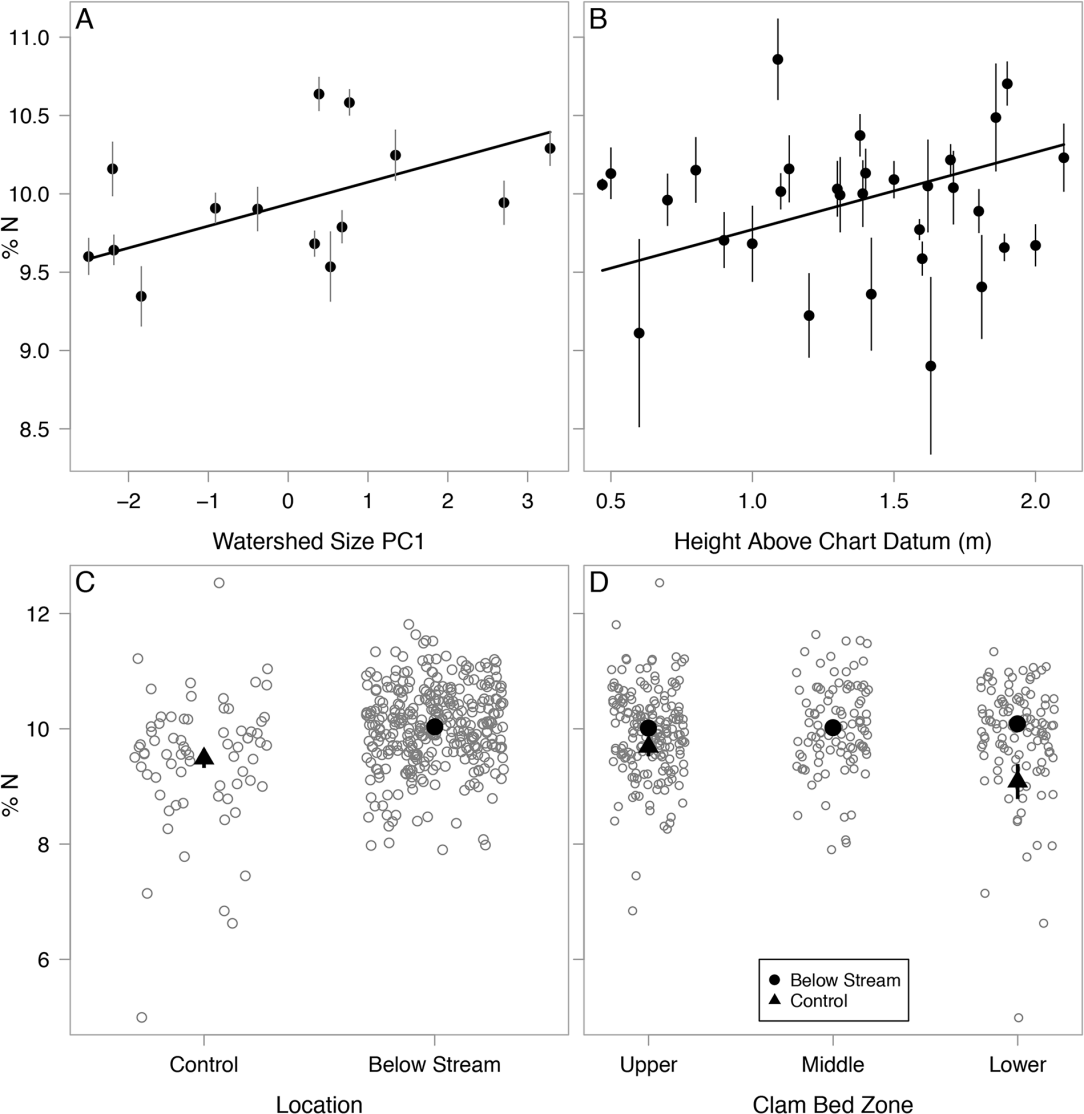
Correlates of soft-shell muscle tissue %N. (A) Watershed size PC1, (B) Height above chart datum, (C) Below stream vs. control locations, and (D) Clam bed zones. Data points in panels A and B represent mean values with standard error bars. Hollow data points in panels C and D represent individual clams; points were dispersed using a jitter function for better visualization. Solid symbols in panels C and D indicate mean values with standard error bars (control locations were not sampled at middle zones). All trend lines represent relationships using intercept and coefficients from multi-model output; thus they represent the relationships for the x-axis variable that accounts for the effects of other variables, rather than fitting the univariate data shown in each graph.

The pseudo- R^2^ of the averaged model was 0.21. Clams below larger watersheds had higher percentages of N in their muscle tissues (Fig 8A). Contrary to our predictions, clams in upper zones contained higher %N in their tissues than their counterparts (Fig 8B). Analysis did not detect an influence from any other covariates including salmon density, temperature, clam size and age. Although zone and location did not have any interaction effects with salmon density or watershed size, clams below streams had higher %N than those in control locations (Fig 8C) and this disparity was most apparent in lower clam beds (Fig 8D). The standardized effects of salmon on %N were negative but highly uncertain at all zone and location levels with confidence intervals crossing 0 (Fig 7B). The effects of watershed size were positive, particularly in upper clam beds and below streams and confidence intervals did not cross 0, with the exception of control locations. Clams higher above chart datum had elevated %N with confidence intervals well above 0 while all remaining covariates had undetectable effects (Fig 7B). The averaged %N model and candidate set are presented in S8 and S9 Tables.

## Discussion

This study demonstrates the complex relationships between terrestrial and marine resource subsidies and traits of sedentary estuarine consumers. It highlights how cross-ecosystem resource linkages can vary both within and across landscapes. Watershed size and salmon density, individual traits, and habitat characteristics described, to varying degrees, stable isotope ratios of soft-shell clam muscle tissue. The effects of watershed size and salmon subsidies on isotope ratios generally decreased from upper to lower zones of clam beds. Clams size and percent nitrogen increased as the size of upstream watersheds increased. However the effect on clam size was only observed in tidal flats below streams and not in control locations. We were surprised to find that upstream salmon density had a negative relationship with clam size, though this negative effect was weaker below streams compared to control locations. To our knowledge, this is the first study to simultaneously test for terrestrial- and salmon-derived subsidies between and within sites across a broad spatial scale.

Watershed size explained isotope ratios and correlated positively with both size and %N of softshell clams. These results support other studies that have demonstrated the importance of terrestrial-derived resource subsidies to estuaries [23,24,50], which can scale directly with watershed size [19,26]. In this case the quantity of terrestrial resource influx into estuaries, as explained by watershed size, appears to be more important than higher-quality pulsed inputs such as salmon-nutrients. Although watershed nutrient exports may be lower quality than salmon or estuarine resources, total energy export from upstream may overwhelm other sources and make it a more influential resource [8,68].

The considerable depletion effect of watershed size on stable isotope ratios in soft-shell clams suggests that terrestrial-derived resources are consumed in proportion to their availability, as also found in a study of Dungeness crabs, *Metacarcinus magister*, [26]. Stream exports are dominated by terrestrial-derived organic material [69], which have low proportions of nitrogen and carbon heavy isotopes. These inputs into estuaries also increase in proportion to watershed size [19,70]. Thus as influx of terrestrial-derived resources increases, soft-shell clams become more ‘terrestrial’ in their isotopic ratios. Although the effects of watershed size on δ^13^C were clear, the effects on δ^15^N were less so, with a strong effect only in lower clam beds. This could be a result of settlement dynamics of particulate organic matter, which could favour deeper individuals [24]. However, as watershed sizes increase, there can also be a shift from heterotrophic nitrogen inputs to autotrophic production in streams, while maintaining a reliance on terrestrial-derived carbon [71]. Therefore, terrestrial-derived nitrogen subsidies could be displaced by freshwater algal nitrogen exports as watershed size increases [51]. Because δ^15^N in stream algae enriches with watershed size [26], and thus becomes more similar to enriched estuarine sources [21], any relationships between watershed size and δ^15^N could be masked.

Clams in estuaries below large watersheds were also larger, with higher percentages of nitrogen in their tissues. Bivalves have the ability to consume terrestrial-derived particulate organic matter directly [49,50]. They may also benefit indirectly, through subsidized abundances of diatoms, bacteria and microphytobenthos [72]. Because growth and %N of soft-shell clams are known to increase with nutrient loading and water flow [73,74], elevated resource imports into estuaries from larger watersheds [19] could allow individuals to grow larger, faster and with higher nitrogen content in tissues [73]. Soft-shell clams are well-suited to these types of resource subsidies, relative to other species, as they can maintain growth at higher nutrient and particulate matter concentrations [75,76]. Although watershed size appears to increase soft-shell %N throughout estuaries, the positive effect on size appears to be limited to the depositional zones below streams. A possible explanation could be sub-optimal habitat limitations to clam growth in control locations as we observed more coarse substrates such as cobble and gravel in these areas. Observed trends in clam size could also be influenced by differences in sediment grain sizes between sampling locations and estuaries, which did not measure in this study. However, clam bed locations below river outlets consisted of sand, mud and fine crushed shell mixtures which do not inhibit growth to the degree of larger substrate sizes [77]. Because growth in bivalves is known to be density dependent, lower clam densities below larger watersheds could also explain the positive correlation between watershed size and clam size [78]. Unfortunately we were unable to properly assess clam densities due to time constraints with low tides, which is a limitation to this study. However qualitative observations did not reveal any noticeable correlations between clam availability and watershed size. It is also possible that more established and mature populations and higher stream flows below large watersheds could hinder larval recruitment success and bias size distributions towards larger individuals [79,80].

Pink salmon density was a strong correlate with soft-shell clam isotope ratios compared to chum salmon. Pink salmon tend to spawn closer to estuaries than chum salmon in our study region, which increases habitat overlap with bivalves. This effect also decreased moving from upper to lower clam bed zones.

Much to our surprise, salmon density had a negative correlation with clam size. One possible explanation is that bivalves require smaller particle sizes such as sand while salmon require coarser gravel for spawning [34], so sites favorable for pink salmon could be less favourable to clams [de 81]. The timing of salmon resource subsides, just before dormant winter periods, could also result in the routing of any energetic benefits from salmon nutrients to metabolic maintenance instead of tissue growth. Salmon can also play a dual role in stream ecosystems as sources of both nutrient subsidies and disturbance [26,82]. Pink salmon spawning in upper reaches of estuaries may exert similar disturbances to bivalves as they disrupt the substrate while digging and defending nests [83,84].

Both size and age of soft-shell clams were strongly correlated with stable isotopes. Few studies consider individual-level traits when using isotopes as an ecological tool and this study underscores the importance of their consideration [26,85,86]. In addition, local habitat conditions can influence isotope ratios, particularly for sedentary organisms such as bivalves. Clams higher in the intertidal were enriched in δ^15^N, likely reflecting a reduction in isotope discrimination as a result of more limited feeding opportunities. Higher estuary temperatures also correlated with more depleted δ^13^C, which was contrary to our expectations. Higher water temperatures upstream could elevate exports of isotopically depleted terrestrial detritus from watersheds as a result of faster decomposition of organic matter. Alternately, this relationship could reflect the positive correlation of temperature with the percentage of alder trees upstream, which we dropped from our analyses (see Methods). Alder trees can provide substantial inputs of isotopically depleted detritus [87,88], which could also deplete soft-shell clam isotope ratios.

As expected, larger clams were found deeper in the intertidal, suggesting higher survival or growth. These clams also had reduced %N in their tissues compared to shallower individuals, contrary to our prediction. Terrestrial-derived nitrogen subsidies could be more concentrated higher in the intertidal and diluted lower down where clams are tidally submerged for longer periods of time. However, because %N of clam tissues is known to increase with growth rates [89], this result may reflect the fact that larger, and thus slower growing clams are concentrated deeper in the intertidal while smaller and faster growing clams dominate shallower locations.

Our work demonstrates the importance of connectivity amongst coastal landscapes and that this connectivity can vary with landscape traits. Our results, and other work, also suggest that the effect of watershed size can broaden food web connectivity, through increased inputs of upstream resources [26,50]. Harding and Reynolds [26] observed increases in Dungeness crab size in response to terrestrial resource influx within the same region, implying these subsidies may have broader effects within estuarine food webs. Animal movement, such as spawning salmon migrations, also provides substantial resource inputs into these ecosystems. Due to the open nature of estuaries, resource subsides have the potential to stabilize these communities, increase productivity, and increase resilience to disturbance and periods of resource scarcity [90,91]. Natural flow regimes are an essential component to the maintenance of subsidy dynamics [92], providing resource linkages and passage for animal movement between terrestrial, freshwater and marine landscapes [93]. These considerations have direct implications for estuarine productivity in intact ecosystems such as the central coast of British Columbia, which faces increasing industrial development pressures that can disrupt discharge regimes and alter resource dynamics [94,95]. Estuaries buffer coastlines and produce resources crucial to coastal First Nations and commercial and recreational fisheries. Recognizing the importance of cross-ecosystem resource linkages in maintaining ecosystems can better enable us to understand how they might respond to human-driven pressures such as resource extraction and climate change [50]. Broader-scale studies such as this can also shed light on how cross-ecosystem processes vary across space and thus can promote realistic resource management and conservation frameworks that acknowledge the inherent heterogeneity in natural systems.

## Supporting Information

S1 Table Model weights of salmon linear models predicting δ^15^N and δ^13^C in soft-shell clam foot muscle tissue. (DOCX)

S2 Table Average coefficient estimates from multi-model analysis of candidate model set for soft-shell clam foot muscle tissue δ^15^N. (DOCX)

S3 Table Candidate model set from multi-model inference of soft-shell clam foot muscle tissue δ^15^N. (DOCX)

S4 Table Average coefficient estimates from multi-model analysis of candidate model set for soft-shell clam foot muscle tissue δ^13^C. (DOCX)

S5 Table Candidate model set from multi-model inference of soft-shell clam foot muscle tissue δ^13^C. (DOCX)

S6 Table Average coefficient estimates from multi-model analysis of candidate model set for soft-shell clam mass. (DOCX)

S7 Table Candidate model set from multi-model inference of soft-shell clam mass. (DOCX)

S8 Table Average coefficient estimates from multi-model analysis of candidate model set for soft-shell clam %N. (DOCX)

S9 Table Candidate model set from multi-model inference of soft-shell clam %N. (DOCX)

S1 Fig Bivariate plot of individual soft-shell clam mass versus shell length. (TIF)
