# Supplementary material for: Location Is Everything: Evaluating the Effects of Terrestrial and Marine Resource Subsidies on an Estuarine Bivalve
Source: PLoS One. 2015 May 18;10(5):e0125167. doi: 10.1371/journal.pone.0125167 (PMC4436346; doi:10.1371/journal.pone.0125167)
Supplement: S1 Table — (DOCX) [file pone.0125167.s001.docx]

**S1 Table. Model weights of salmon linear models predicting δ^15^N and δ^13^C in soft-shell clam foot muscle tissue.**

| **δ^15^N Salmon Model** | **Weight** |  | **δ^13^C Salmon Model** | **Weight** |
| --- | --- | --- | --- | --- |
| Mean pink 2006-07 | 0.82 |  | Mean pink 2006-07 | 0.89 |
| Mean pink 2006-08 | 0.18 |  | Mean pink 2006-09 | 0.11 |
| Mean pink 2006-09 | 6.94E-08 |  | Mean pink 2006-08 | 3.14E-06 |
| Year prior pink | 1.25E-12 |  | Mean salmon 2006-10 | 1.91E-06 |
| Mean salmon 2006-10 | 6.00E-13 |  | Mean salmon 2006-09 | 1.47E-06 |
| Mean salmon 2006-09 | 1.14E-13 |  | Mean salmon 2006-07 | 7.79E-09 |
| Mean salmon 2006-08 | 1.13E-16 |  | Mean salmon 2006-08 | 6.06E-10 |
| Mean chum 2006-09 | 2.36E-17 |  | Year prior pink | 3.69E-10 |
| Year prior salmon | 2.19E-17 |  | Mean chum 2006-09 | 2.64E-10 |
| Mean salmon 2006-07 | 1.39E-18 |  | Mean chum 2006-07 | 1.20E-10 |
| Mean chum 2006-08 | 1.88E-20 |  | Year prior salmon | 1.11E-10 |
| Year prior chum | 2.32E-21 |  | Mean chum 2006-08 | 8.81E-11 |
| Mean chum 2006-07 | 3.48E-22 |  | Year prior chum | 8.19E-11 |

Mean salmon metrics include combined pink and chum densities. All pink, chum and salmon metrics are mean values across years indicated in (kg/m^2^).
