# Supplementary material for: Location Is Everything: Evaluating the Effects of Terrestrial and Marine Resource Subsidies on an Estuarine Bivalve
Source: PLoS One. 2015 May 18;10(5):e0125167. doi: 10.1371/journal.pone.0125167 (PMC4436346; doi:10.1371/journal.pone.0125167)
Supplement: S2 Table — (DOCX) [file pone.0125167.s002.docx]

**S2 Table. Average coefficient estimates from multi-model analysis of candidate model set for soft-shell clam foot muscle tissue δ^15^N.**

| **Covariate** | **Estimate** | **SE** | **Lower CI** | **Upper CI** | **RVI** |
| --- | --- | --- | --- | --- | --- |
| Intercept | 8.91 | 0.08 | 8.75 | 9.06 |  |
| Age | 0.07 | 0.01 | 0.05 | 0.09 | 1.00 |
| Lower | -0.08 | 0.07 | -0.22 | 0.05 | 1.00 |
| Salmon*Lower | -1.01 | 0.36 | -1.72 | -0.29 | 1.00 |
| Salmon | 2.05 | 0.75 | 0.58 | 3.53 | 1.00 |
| Mass | 0.00 | 0.00 | 0.00 | 0.01 | 1.00 |
| Depth | 0.17 | 0.07 | 0.03 | 0.30 | 0.96 |
| WS | -0.02 | 0.05 | -0.12 | 0.09 | 0.94 |
| WS*Lower | -0.06 | 0.03 | -0.11 | -0.01 | 0.88 |
| Middle | -0.07 | 0.05 | -0.17 | 0.04 | 0.74 |
| Salmon*Middle | -0.61 | 0.38 | -1.35 | 0.14 | 0.42 |
| WS*Middle | 0.04 | 0.03 | -0.02 | 0.09 | 0.29 |
| Below Stream | 0.00 | 0.05 | -0.10 | 0.10 | 0.28 |
| Temperature | -0.02 | 0.04 | -0.09 | 0.05 | 0.19 |
| Salmon*Below Stream | -0.52 | 0.42 | -1.34 | 0.31 | 0.09 |
| WS*Below Stream | 0.02 | 0.03 | -0.04 | 0.08 | 0.03 |

The coefficient for below stream locations is relative to control locations; the coefficients for middle and lower zones are relative to the upper zone. SE= standard error, Lower CI and Upper CI are 95% confidence intervals and RVI = relative variable importance, which is the sum of weights for all models in which each covariate is present.
