# Supplementary material for: Location Is Everything: Evaluating the Effects of Terrestrial and Marine Resource Subsidies on an Estuarine Bivalve
Source: PLoS One. 2015 May 18;10(5):e0125167. doi: 10.1371/journal.pone.0125167 (PMC4436346; doi:10.1371/journal.pone.0125167)
Supplement: S3 Table — (DOCX) [file pone.0125167.s003.docx]

**S3 Table. Candidate model set (those from the global model set with a ΔAICc less than 4.0) from multi-model inference of soft-shell clam foot muscle tissue δ^15^N.**

| **δ^15^N Models** | ***k*** | **logLik** | **AICc** | **ΔAICc** | **Weight** |
| --- | --- | --- | --- | --- | --- |
| Age+ Depth+ Mass+ Salmon*Middle+ Salmon*Lower+ WS*Lower | 14 | -168.21 | 365.52 | 0.00 | 0.11 |
| Age+ Depth+ Mass+ Salmon*Lower+ WS*Lower | 12 | -170.42 | 365.65 | 0.13 | 0.10 |
| Age+ Middle+ Depth+ Mass+ Salmon*Lower+ WS*Lower | 13 | -169.70 | 366.34 | 0.83 | 0.07 |
| Age+ Depth+ Mass+ Salmon*Middle+ WS*Middle+ Salmon*Lower+ WS*Lower | 15 | -167.60 | 366.45 | 0.94 | 0.07 |
| Age+ Depth+ Mass+ WS*Middle+ Salmon*Lower+ WS*Lower | 14 | -168.75 | 366.59 | 1.08 | 0.06 |
| Age+ Depth+ Temperature+ Mass+ Salmon*Middle+ Salmon*Lower+ WS*Lower | 15 | -168.01 | 367.29 | 1.77 | 0.04 |
| Age+ Depth+ Temperature+ Mass+ Salmon*Lower+ WS*Lower | 13 | -170.22 | 367.39 | 1.88 | 0.04 |
| Age+ Below Stream+ Depth+ Mass+ Salmon*Middle+ Salmon*Lower+ WS*Lower | 15 | -168.20 | 367.66 | 2.15 | 0.04 |
| Age+ Below Stream+ Depth+ Mass+ Salmon*Lower+ WS*Lower | 13 | -170.39 | 367.72 | 2.21 | 0.03 |
| Age+ Depth+ Mass+ Salmon*Below Stream+ Salmon*Lower+ WS*Lower | 14 | -169.42 | 367.95 | 2.43 | 0.03 |
| Age+ Depth+ Mass+ WS*Middle+ Salmon*Lower | 13 | -170.50 | 367.95 | 2.44 | 0.03 |
| Age+ Middle+ Depth+ Temperature+ Mass+ Salmon*Lower+ WS*Lower | 14 | -169.53 | 368.16 | 2.64 | 0.03 |
| Age+ Depth+ Temperature+ Mass+ Salmon*Middle+ WS*Middle+ Salmon*Lower+ WS*Lower | 16 | -167.41 | 368.25 | 2.73 | 0.03 |
| Age+ Depth+ Mass+ Salmon*Middle+ WS*Middle+ Salmon*Lower | 14 | -169.60 | 368.31 | 2.79 | 0.03 |
| Age+ Depth+ Temperature+ Mass+ WS*Middle+ Salmon*Lower+ WS*Lower | 15 | -168.58 | 368.42 | 2.91 | 0.02 |
| Age+ Below Stream+ Middle+ Depth+ Mass+ Salmon*Lower+ WS*Lower | 14 | -169.69 | 368.48 | 2.97 | 0.02 |
| Age+ Below Stream+ Depth+ Mass+ Salmon*Middle+ WS*Middle+ Salmon*Lower+ WS*Lower | 16 | -167.60 | 368.62 | 3.11 | 0.02 |
| Age+ Depth+ Mass+ Salmon*Lower | 10 | -174.06 | 368.69 | 3.17 | 0.02 |
| Age+ Depth+ Mass+ Salmon*Middle+ Salmon*Lower | 12 | -171.95 | 368.71 | 3.19 | 0.02 |
| Age+ Middle+ Depth+ Mass+ Salmon*Below Stream+ Salmon*Lower+ WS*Lower | 15 | -168.75 | 368.75 | 3.24 | 0.02 |
| Age+ Below Stream+ Depth+ Mass+ WS*Middle+ Salmon*Lower+ WS*Lower | 15 | -168.75 | 368.75 | 3.24 | 0.02 |
| Age+ Mass+ Salmon*Middle+ Salmon*Lower+ WS*Lower | 13 | -170.91 | 368.78 | 3.26 | 0.02 |
| Age+ Depth+ Mass+ Salmon*Below Stream+ Salmon*Middle+ Salmon*Lower+ WS*Lower | 16 | -167.72 | 368.88 | 3.36 | 0.02 |
| Age+ Depth+ Mass+ Salmon*Below Stream+ WS*Middle+ Salmon*Lower+ WS*Lower | 16 | -167.95 | 369.34 | 3.82 | 0.02 |
| Age+ Depth+ Mass+ WS*Below Stream+ Salmon*Lower+ WS*Lower | 14 | -170.13 | 369.35 | 3.83 | 0.02 |
| Age+ Middle+ Depth+ Mass+ Salmon*Lower | 11 | -173.33 | 369.35 | 3.84 | 0.02 |
| Age+ Below Stream+ Depth+ Temperature+ Mass+ Salmon*Middle+ Salmon*Lower+ WS*Lower | 16 | -168.01 | 369.45 | 3.94 | 0.01 |
| Age+ Below Stream+ Depth+ Temperature+ Mass+ Salmon*Lower+ WS*Lower | 14 | -170.18 | 369.47 | 3.95 | 0.01 |
| Age+ Middle+ Mass+ Salmon*Lower+ WS*Lower | 12 | -172.34 | 369.48 | 3.97 | 0.01 |
| Age+ Depth+ Mass+ WS*Below Stream+ Salmon*Middle+ Salmon*Lower+ WS*Lower | 16 | -168.04 | 369.51 | 3.99 | 0.01 |

*k* = number of model parameters, logLik = model log likelihood, ΔAICc = change in AICc score from top model, weight = AICc model weight.
