# Supplementary material for: Location Is Everything: Evaluating the Effects of Terrestrial and Marine Resource Subsidies on an Estuarine Bivalve
Source: PLoS One. 2015 May 18;10(5):e0125167. doi: 10.1371/journal.pone.0125167 (PMC4436346; doi:10.1371/journal.pone.0125167)
Supplement: S4 Table — (DOCX) [file pone.0125167.s004.docx]

**S4 Table. Average coefficient estimates from multi-model analysis of candidate model set for soft-shell clam foot muscle tissue δ^13^C.**

| **Covariate** | **Estimate** | **SE** | **Lower CI** | **Upper CI** | **RVI** |
| --- | --- | --- | --- | --- | --- |
| Intercept | -18.32 | 0.07 | -18.46 | -18.19 |  |
| Age | 0.07 | 0.01 | 0.05 | 0.09 | 1.00 |
| Below Stream | -0.20 | 0.05 | -0.29 | -0.11 | 1.00 |
| WS*Below Stream | -0.09 | 0.03 | -0.15 | -0.03 | 1.00 |
| Lower | -0.12 | 0.05 | -0.21 | -0.03 | 1.00 |
| Temperature | -0.10 | 0.03 | -0.16 | -0.04 | 1.00 |
| Salmon | 1.85 | 0.68 | 0.51 | 3.18 | 1.00 |
| Mass | 0.00 | 0.00 | 0.00 | 0.01 | 1.00 |
| WS | -0.12 | 0.05 | -0.21 | -0.02 | 1.00 |
| WS*Lower | -0.06 | 0.02 | -0.10 | -0.01 | 0.96 |
| Salmon*Lower | -0.67 | 0.30 | -1.26 | -0.07 | 0.95 |
| Middle | 0.03 | 0.05 | -0.06 | 0.12 | 0.53 |
| WS*Middle | 0.04 | 0.02 | -0.01 | 0.09 | 0.30 |
| Depth | -0.04 | 0.06 | -0.16 | 0.08 | 0.21 |
| Salmon*Below Stream | 0.16 | 0.38 | -0.59 | 0.90 | 0.19 |
| Salmon*Middle | -0.36 | 0.34 | -1.04 | 0.32 | 0.12 |

The coefficient for below stream locations is relative to control locations; the coefficients for middle and lower zones are relative to the upper zone. Table headings described in Table S2.
