# Supplementary material for: Location Is Everything: Evaluating the Effects of Terrestrial and Marine Resource Subsidies on an Estuarine Bivalve
Source: PLoS One. 2015 May 18;10(5):e0125167. doi: 10.1371/journal.pone.0125167 (PMC4436346; doi:10.1371/journal.pone.0125167)
Supplement: S5 Table — (DOCX) [file pone.0125167.s005.docx]

**S5 Table. Candidate model set (those from the global model set with a ΔAICc less than 4.0) from multi-model inference of soft-shell clam foot muscle tissue δ^13^C.**

| **δ^13^C Models** | ***k*** | **logLik** | **AICc** | **ΔAICc** | **Weight** |
| --- | --- | --- | --- | --- | --- |
| Age+ Temperature+ Mass+ WS*Below Stream+ Salmon*Lower+ WS*Lower | 14 | -127.74 | 284.57 | 0.00 | 0.21 |
| Age+ Temperature+ Mass+ WS*Below Stream+ WS*Middle+ Salmon*Lower+ WS*Lower | 16 | -126.21 | 285.84 | 1.27 | 0.11 |
| Age+ Middle+ Temperature+ Mass+ WS*Below Stream+ Salmon*Lower+ WS*Lower | 15 | -127.47 | 286.21 | 1.63 | 0.09 |
| Age+ Depth+ Temperature+ Mass+ WS*Below Stream+ Salmon*Lower+ WS*Lower | 15 | -127.48 | 286.21 | 1.64 | 0.09 |
| Age+ Temperature+ Mass+ Salmon*Below Stream+ WS*Below Stream+ Salmon*Lower+ WS*Lower | 15 | -127.67 | 286.60 | 2.03 | 0.08 |
| Age+ Temperature+ Mass+ WS*Below Stream+ Salmon*Middle+ Salmon*Lower+ WS*Lower | 16 | -126.77 | 286.98 | 2.41 | 0.06 |
| Age+ Temperature+ Mass+ WS*Below Stream+ Salmon*Middle+ WS*Middle+ Salmon*Lower+ WS*Lower | 17 | -125.76 | 287.13 | 2.55 | 0.06 |
| Age+ Temperature+ Salmon+ Mass+ WS*Below Stream+ WS*Lower | 13 | -130.26 | 287.48 | 2.90 | 0.05 |
| Age+ Depth+ Temperature+ Mass+ WS*Below Stream+ WS*Middle+ Salmon*Lower+ WS*Lower | 17 | -126.06 | 287.74 | 3.16 | 0.04 |
| Age+ Temperature+ Mass+ Salmon*Below Stream+ WS*Below Stream+ WS*Middle+ Salmon*Lower+ WS*Lower | 17 | -126.11 | 287.83 | 3.25 | 0.04 |
| Age+ Temperature+ Mass+ WS*Below Stream+ WS*Middle+ Salmon*Lower | 15 | -128.29 | 287.84 | 3.27 | 0.04 |
| Age+ Depth+ Temperature+ Mass+ Salmon*Below Stream+ WS*Below Stream+ Salmon*Lower+ WS*Lower | 16 | -127.33 | 288.10 | 3.52 | 0.04 |
| Age+ Middle+ Depth+ Temperature+ Mass+ WS*Below Stream+ Salmon*Lower+ WS*Lower | 16 | -127.35 | 288.12 | 3.55 | 0.04 |
| Age+ Middle+ Temperature+ Mass+ Salmon*Below Stream+ WS*Below Stream+ Salmon*Lower+ WS*Lower | 16 | -127.39 | 288.21 | 3.64 | 0.03 |

Table headings described in Table S3.
