# Supplementary material for: Location Is Everything: Evaluating the Effects of Terrestrial and Marine Resource Subsidies on an Estuarine Bivalve
Source: PLoS One. 2015 May 18;10(5):e0125167. doi: 10.1371/journal.pone.0125167 (PMC4436346; doi:10.1371/journal.pone.0125167)
Supplement: S6 Table — (DOCX) [file pone.0125167.s006.docx]

**S6 Table. Average coefficient estimates from multi-model analysis of candidate model set for soft-shell clam mass.**

| **Covariate** | **Estimate** | **SE** | **Lower CI** | **Upper CI** | **RVI** |
| --- | --- | --- | --- | --- | --- |
| Intercept | 39.67 | 3.11 | 33.59 | 45.76 |  |
| Age | 4.74 | 0.31 | 4.13 | 5.35 | 1.00 |
| Below Stream | -1.73 | 1.99 | -5.63 | 2.16 | 1.00 |
| Salmon*Below Stream | 65.08 | 15.55 | 34.61 | 95.55 | 1.00 |
| WS*Below Stream | 6.13 | 1.21 | 3.76 | 8.50 | 1.00 |
| Middle | 6.89 | 2.05 | 2.87 | 10.92 | 1.00 |
| Lower | 8.58 | 2.77 | 3.16 | 14.01 | 1.00 |
| Depth | -10.45 | 2.97 | -16.27 | -4.62 | 1.00 |
| Salmon | -35.15 | 29.89 | -93.75 | 23.44 | 1.00 |
| WS | 2.27 | 2.08 | -1.80 | 6.34 | 1.00 |
| WS*Lower | 1.43 | 1.13 | -0.79 | 3.65 | 0.43 |
| Salmon*Lower | 16.45 | 14.31 | -11.60 | 44.49 | 0.40 |
| WS*Middle | 0.39 | 1.04 | -1.65 | 2.42 | 0.18 |
| Salmon*Middle | -2.20 | 13.18 | -28.03 | 23.63 | 0.17 |
| Temperature | 0.22 | 1.44 | -2.60 | 3.04 | 0.17 |

The coefficient for below stream locations is relative to control locations; the coefficients for middle and lower zones are relative to the upper zone. Table headings described in Table S2.
