# Supplementary material for: Location Is Everything: Evaluating the Effects of Terrestrial and Marine Resource Subsidies on an Estuarine Bivalve
Source: PLoS One. 2015 May 18;10(5):e0125167. doi: 10.1371/journal.pone.0125167 (PMC4436346; doi:10.1371/journal.pone.0125167)
Supplement: S7 Table — (DOCX) [file pone.0125167.s007.docx]

**S7 Table. Candidate model set (those from the global model set with a ΔAICc less than 4.0) from multi-model inference of soft-shell clam mass.**

| **Mass Models** | ***k*** | **logLik** | **AICc** | **ΔAICc** | **Weight** |
| --- | --- | --- | --- | --- | --- |
| Age+ Middle+ Lower+ Depth+ Salmon*Below Stream+ WS*Below Stream | 14 | -1644.03 | 3317.16 | 0.00 | 0.17 |
| Age+ Middle+ Depth+ Salmon*Below Stream+ WS*Below Stream+ WS*Lower | 15 | -1643.35 | 3317.97 | 0.80 | 0.12 |
| Age+ Middle+ Depth+ Salmon*Below Stream+ WS*Below Stream+ Salmon*Lower | 15 | -1643.45 | 3318.17 | 1.00 | 0.11 |
| Age+ Middle+ Depth+ Salmon*Below Stream+ WS*Below Stream+ Salmon*Lower+ WS*Lower | 16 | -1642.52 | 3318.46 | 1.30 | 0.09 |
| Age+ Lower+ Depth+ Salmon*Below Stream+ WS*Below Stream+ WS*Middle | 15 | -1644.00 | 3319.26 | 2.10 | 0.06 |
| Age+ Lower+ Depth+ Salmon*Below Stream+ WS*Below Stream+ Salmon*Middle | 15 | -1644.00 | 3319.26 | 2.10 | 0.06 |
| Age+ Middle+ Lower+ Depth+ Temperature+ Salmon*Below Stream+ WS*Below Stream | 15 | -1644.02 | 3319.30 | 2.13 | 0.06 |
| Age+ Depth+ Salmon*Below Stream+ WS*Below Stream+ WS*Middle+ WS*Lower | 16 | -1643.17 | 3319.76 | 2.60 | 0.05 |
| Age+ Depth+ Salmon*Below Stream+ WS*Below Stream+ Salmon*Middle+ WS*Lower | 16 | -1643.29 | 3320.02 | 2.86 | 0.04 |
| Age+ Middle+ Depth+ Temperature+ Salmon*Below Stream+ WS*Below Stream+ WS*Lower | 16 | -1643.34 | 3320.11 | 2.94 | 0.04 |
| Age+ Middle+ Depth+ Temperature+ Salmon*Below Stream+ WS*Below Stream+ Salmon*Lower | 16 | -1643.44 | 3320.31 | 3.14 | 0.04 |
| Age+ Depth+ Salmon*Below Stream+ WS*Below Stream+ WS*Middle+ Salmon*Lower | 16 | -1643.44 | 3320.32 | 3.15 | 0.04 |
| Age+ Depth+ Salmon*Below Stream+ WS*Below Stream+ Salmon*Middle+ Salmon*Lower | 16 | -1643.45 | 3320.34 | 3.18 | 0.04 |
| Age+ Depth+ Salmon*Below Stream+ WS*Below Stream+ WS*Middle+ Salmon*Lower+ WS*Lower | 17 | -1642.37 | 3320.36 | 3.20 | 0.04 |
| Age+ Middle+ Depth+ Temperature+ Salmon*Below Stream+ WS*Below Stream+ Salmon*Lower+ WS*Lower | 17 | -1642.50 | 3320.62 | 3.45 | 0.03 |
| Age+ Depth+ Salmon*Below Stream+ WS*Below Stream+ Salmon*Middle+ Salmon*Lower+ WS*Lower | 17 | -1642.52 | 3320.65 | 3.48 | 0.03 |

Table headings described in Table S3.
