# Supplementary material for: Location Is Everything: Evaluating the Effects of Terrestrial and Marine Resource Subsidies on an Estuarine Bivalve
Source: PLoS One. 2015 May 18;10(5):e0125167. doi: 10.1371/journal.pone.0125167 (PMC4436346; doi:10.1371/journal.pone.0125167)
Supplement: S8 Table — (DOCX) [file pone.0125167.s008.docx]

**S8 Table. Average coefficient estimates from multi-model analysis of centered candidate model set for soft-shell clam %N.**

| **Covariate** | **Estimate** | **SE** | **Lower CI** | **Upper CI** | **RVI** |
| --- | --- | --- | --- | --- | --- |
| Intercept | 9.96 | 0.08 | 9.79 | 10.12 |  |
| Below Stream | 0.48 | 0.14 | 0.20 | 0.76 | 1.00 |
| Depth | 0.49 | 0.13 | 0.24 | 0.74 | 1.00 |
| WS | 0.14 | 0.06 | 0.03 | 0.25 | 1.00 |
| Lower | 0.27 | 0.11 | 0.04 | 0.49 | 0.99 |
| Middle | 0.11 | 0.09 | -0.06 | 0.29 | 0.58 |
| Salmon | -0.73 | 0.73 | -2.15 | 0.69 | 0.51 |
| WS*Below Stream | 0.13 | 0.09 | -0.05 | 0.30 | 0.49 |
| WS*Lower | -0.06 | 0.04 | -0.15 | 0.03 | 0.46 |
| WS*Middle | -0.05 | 0.05 | -0.14 | 0.04 | 0.17 |
| Salmon*Lower | 0.77 | 0.65 | -0.51 | 2.05 | 0.16 |
| Mass | 0.00 | 0.00 | 0.00 | 0.00 | 0.15 |
| Salmon*Middle | 0.95 | 0.66 | -0.34 | 2.24 | 0.14 |
| Age | 0.00 | 0.01 | -0.02 | 0.03 | 0.13 |
| Temperature | 0.01 | 0.04 | -0.06 | 0.07 | 0.13 |
| Salmon*Below Stream | 0.19 | 1.21 | -2.17 | 2.55 | 0.05 |

The coefficient for below stream locations is relative to control locations; the coefficients for middle and lower zones are relative to the upper zone. Table headings described in Table S2.
