# Supplementary material for: Location Is Everything: Evaluating the Effects of Terrestrial and Marine Resource Subsidies on an Estuarine Bivalve
Source: PLoS One. 2015 May 18;10(5):e0125167. doi: 10.1371/journal.pone.0125167 (PMC4436346; doi:10.1371/journal.pone.0125167)
Supplement: S9 Table — (DOCX) [file pone.0125167.s009.docx]

**S9 Table. Candidate model set (those from the global model set with a ΔAICc less than 4.0) from multi-model inference of soft-shell clam %N.**

| **%N Models** | ***k*** | **logLik** | **AICc** | **ΔAICc** | **Weight** |
| --- | --- | --- | --- | --- | --- |
| Below Stream+ Lower+ Depth+ WS | 9 | -407.80 | 834.07 | 0.00 | 0.029 |
| Lower+ Depth+ WS*Below Stream | 10 | -406.78 | 834.13 | 0.06 | 0.028 |
| Below Stream+ Depth+ WS+ WS*Lower | 10 | -406.95 | 834.46 | 0.39 | 0.024 |
| Below Stream+ Middle+ Lower+ Depth+ WS | 10 | -406.98 | 834.53 | 0.45 | 0.023 |
| Depth+ WS*Below Stream+ WS*Lower | 11 | -405.97 | 834.63 | 0.56 | 0.022 |
| Middle+ Lower+ Depth+ WS*Below Stream | 11 | -405.99 | 834.66 | 0.59 | 0.021 |
| Below Stream+ Middle+ Depth+ WS*Lower | 11 | -406.16 | 835.02 | 0.94 | 0.018 |
| Depth+ WS*Below Stream+ WS*Middle+ WS*Lower | 13 | -404.04 | 835.03 | 0.96 | 0.018 |
| Below Stream+ Lower+ Depth+ Salmon+ WS | 10 | -407.28 | 835.13 | 1.06 | 0.017 |
| Lower+ Depth+ Salmon+ WS*Below Stream | 11 | -406.25 | 835.19 | 1.12 | 0.016 |
| Middle+ Depth+ WS*Below Stream+ WS*Lower | 12 | -405.22 | 835.25 | 1.18 | 0.016 |
| Below Stream+ Middle+ Lower+ Depth+ Salmon+ WS | 11 | -406.36 | 835.41 | 1.34 | 0.015 |
| Below Stream+ Depth+ Salmon+ WS*Lower | 11 | -406.40 | 835.48 | 1.41 | 0.014 |
| Below Stream+ Depth+ WS*Middle+ WS*Lower | 12 | -405.36 | 835.53 | 1.45 | 0.014 |
| Middle+ Lower+ Depth+ Salmon+ WS*Below Stream | 12 | -405.37 | 835.55 | 1.48 | 0.014 |
| Depth+ Salmon+ WS*Below Stream+ WS*Lower | 12 | -405.42 | 835.65 | 1.58 | 0.013 |
| Depth+ WS*Below Stream+ Salmon*Middle+ Salmon*Lower | 14 | -403.33 | 835.75 | 1.68 | 0.012 |
| Below Stream+ Lower+ Depth+ Mass+ WS | 10 | -407.63 | 835.83 | 1.76 | 0.012 |
| Below Stream+ Middle+ Depth+ Salmon+ WS*Lower | 12 | -405.53 | 835.87 | 1.80 | 0.012 |
| Below Stream+ Depth+ WS+ Salmon*Middle+ Salmon*Lower | 13 | -404.46 | 835.87 | 1.80 | 0.012 |
| Depth+ Salmon+ WS*Below Stream+ WS*Middle+ WS*Lower | 14 | -403.43 | 835.95 | 1.88 | 0.011 |
| Lower+ Depth+ Mass+ WS*Below Stream | 11 | -406.68 | 836.05 | 1.98 | 0.011 |
| Age+ Below Stream+ Lower+ Depth+ WS | 10 | -407.76 | 836.08 | 2.01 | 0.010 |
| Depth+ WS*Below Stream+ WS*Middle | 12 | -405.64 | 836.09 | 2.02 | 0.010 |
| Middle+ Depth+ Salmon+ WS*Below Stream+ WS*Lower | 13 | -404.58 | 836.11 | 2.03 | 0.010 |
| Below Stream+ Depth+ Mass+ WS*Lower | 11 | -406.74 | 836.16 | 2.09 | 0.010 |
| Age+ Lower+ Depth+ WS*Below Stream | 11 | -406.74 | 836.17 | 2.10 | 0.010 |
| Below Stream+ Lower+ Depth+ Temperature+ WS | 10 | -407.80 | 836.17 | 2.10 | 0.010 |
| Lower+ Depth+ Temperature+ WS*Below Stream | 11 | -406.78 | 836.24 | 2.17 | 0.010 |
| Below Stream+ Depth+ WS+ Salmon*Lower | 11 | -406.80 | 836.28 | 2.21 | 0.010 |
| Below Stream+ Lower+ Depth+ WS*Middle | 11 | -406.80 | 836.29 | 2.22 | 0.009 |
| Below Stream+ Lower+ Depth+ WS+ Salmon*Middle | 12 | -405.75 | 836.31 | 2.24 | 0.009 |
| Depth+ WS*Below Stream+ Salmon*Lower | 12 | -405.76 | 836.33 | 2.26 | 0.009 |
| Lower+ Depth+ WS*Below Stream+ Salmon*Middle | 13 | -404.69 | 836.33 | 2.26 | 0.009 |
| Below Stream+ Depth+ Salmon+ WS*Middle+ WS*Lower | 13 | -404.74 | 836.43 | 2.36 | 0.009 |
| Age+ Below Stream+ Depth+ WS*Lower | 11 | -406.89 | 836.46 | 2.39 | 0.009 |
| Below Stream+ Middle+ Lower+ Depth+ Mass+ WS | 11 | -406.89 | 836.48 | 2.40 | 0.009 |
| Depth+ Mass+ WS*Below Stream+ WS*Lower | 12 | -405.84 | 836.50 | 2.43 | 0.009 |
| Below Stream+ Depth+ Salmon*Middle+ WS*Lower | 13 | -404.79 | 836.54 | 2.47 | 0.008 |
| Below Stream+ Depth+ Temperature+ WS*Lower | 11 | -406.94 | 836.57 | 2.50 | 0.008 |
| Age+ Below Stream+ Middle+ Lower+ Depth+ WS | 11 | -406.95 | 836.59 | 2.52 | 0.008 |
| Below Stream+ Middle+ Lower+ Depth+ Temperature+ WS | 11 | -406.98 | 836.64 | 2.57 | 0.008 |
| Age+ Depth+ WS*Below Stream+ WS*Lower | 12 | -405.92 | 836.65 | 2.58 | 0.008 |
| Depth+ WS*Below Stream+ Salmon*Middle+ WS*Lower | 14 | -403.78 | 836.66 | 2.59 | 0.008 |
| Below Stream+ Middle+ Depth+ WS+ Salmon*Lower | 12 | -405.93 | 836.68 | 2.61 | 0.008 |
| Middle+ Lower+ Depth+ Mass+ WS*Below Stream | 12 | -405.95 | 836.71 | 2.64 | 0.008 |
| Age+ Middle+ Lower+ Depth+ WS*Below Stream | 12 | -405.96 | 836.74 | 2.67 | 0.008 |
| Depth+ Temperature+ WS*Below Stream+ WS*Lower | 12 | -405.97 | 836.75 | 2.68 | 0.008 |
| Middle+ Lower+ Depth+ Temperature+ WS*Below Stream | 12 | -405.99 | 836.79 | 2.72 | 0.007 |
| Depth+ WS*Below Stream+ Salmon*Middle+ Salmon*Lower+ WS*Lower | 15 | -402.76 | 836.79 | 2.72 | 0.007 |
| Middle+ Depth+ WS*Below Stream+ Salmon*Lower | 13 | -404.92 | 836.80 | 2.73 | 0.007 |
| Depth+ WS*Below Stream | 9 | -409.17 | 836.80 | 2.73 | 0.007 |
| Below Stream+ Depth+ Salmon*Middle+ Salmon*Lower+ WS*Lower | 14 | -403.85 | 836.80 | 2.73 | 0.007 |
| Below Stream+ Depth+ WS | 8 | -410.26 | 836.90 | 2.83 | 0.007 |
| Below Stream+ Lower+ Depth+ Salmon+ Mass+ WS | 11 | -407.12 | 836.92 | 2.85 | 0.007 |
| Below Stream+ Middle+ Depth+ Mass+ WS*Lower | 12 | -406.06 | 836.92 | 2.85 | 0.007 |
| Depth+ Mass+ WS*Below Stream+ WS*Middle+ WS*Lower | 14 | -403.94 | 836.99 | 2.91 | 0.007 |
| Depth+ WS*Below Stream+ Salmon*Middle+ WS*Middle+ WS*Lower | 15 | -402.87 | 837.00 | 2.93 | 0.007 |
| Age+ Depth+ WS*Below Stream+ WS*Middle+ WS*Lower | 14 | -403.96 | 837.02 | 2.95 | 0.007 |
| Lower+ Depth+ Salmon+ WS*Below Stream+ WS*Middle | 13 | -405.04 | 837.02 | 2.95 | 0.007 |
| Age+ Below Stream+ Middle+ Depth+ WS*Lower | 12 | -406.13 | 837.07 | 3.00 | 0.006 |
| Below Stream+ Depth+ Salmon*Lower+ WS*Lower | 12 | -406.13 | 837.07 | 3.00 | 0.006 |
| Age+ Below Stream+ Lower+ Depth+ Salmon+ WS | 11 | -407.20 | 837.10 | 3.02 | 0.006 |
| Below Stream+ Lower+ Depth+ Temperature+ Salmon+ WS | 11 | -407.22 | 837.13 | 3.06 | 0.006 |
| Lower+ Depth+ Salmon+ Mass+ WS*Below Stream | 12 | -406.16 | 837.13 | 3.06 | 0.006 |
| Below Stream+ Middle+ Depth+ Temperature+ WS*Lower | 12 | -406.16 | 837.14 | 3.07 | 0.006 |
| Lower+ Depth+ Salmon*Below Stream+ WS*Below Stream | 12 | -406.17 | 837.15 | 3.08 | 0.006 |
| Age+ Lower+ Depth+ Salmon+ WS*Below Stream | 12 | -406.18 | 837.18 | 3.11 | 0.006 |
| Depth+ Temperature+ WS*Below Stream+ WS*Middle+ WS*Lower | 14 | -404.04 | 837.18 | 3.11 | 0.006 |
| Lower+ Depth+ Temperature+ Salmon+ WS*Below Stream | 12 | -406.20 | 837.21 | 3.14 | 0.006 |
| Below Stream+ Lower+ Depth+ Salmon+ WS*Middle | 12 | -406.20 | 837.22 | 3.15 | 0.006 |
| Below Stream+ Depth+ Salmon+ Mass+ WS*Lower | 12 | -406.20 | 837.22 | 3.15 | 0.006 |
| Depth+ WS*Below Stream+ Salmon*Lower+ WS*Lower | 13 | -405.14 | 837.23 | 3.15 | 0.006 |
| Lower+ Depth+ WS+ Salmon*Below Stream | 11 | -407.28 | 837.25 | 3.17 | 0.006 |
| Middle+ Depth+ Mass+ WS*Below Stream+ WS*Lower | 13 | -405.16 | 837.28 | 3.21 | 0.006 |
| Age+ Middle+ Depth+ WS*Below Stream+ WS*Lower | 13 | -405.19 | 837.33 | 3.26 | 0.006 |
| Below Stream+ Depth+ Mass+ WS*Middle+ WS*Lower | 13 | -405.19 | 837.33 | 3.26 | 0.006 |
| Middle+ Depth+ Temperature+ WS*Below Stream+ WS*Lower | 13 | -405.22 | 837.39 | 3.32 | 0.005 |
| Below Stream+ Middle+ Lower+ Depth+ Salmon+ Mass+ WS | 12 | -406.29 | 837.40 | 3.33 | 0.005 |
| Depth+ WS*Below Stream+ Salmon*Middle+ WS*Middle+ Salmon*Lower+ WS*Lower | 16 | -402.00 | 837.42 | 3.35 | 0.005 |
| Age+ Below Stream+ Depth+ Salmon+ WS*Lower | 12 | -406.30 | 837.42 | 3.35 | 0.005 |
| Age+ Below Stream+ Middle+ Lower+ Depth+ Salmon+ WS | 12 | -406.31 | 837.44 | 3.37 | 0.005 |
| Below Stream+ Middle+ Lower+ Depth+ Temperature+ Salmon+ WS | 12 | -406.32 | 837.45 | 3.38 | 0.005 |
| Below Stream+ Depth+ Temperature+ Salmon+ WS*Lower | 12 | -406.34 | 837.49 | 3.42 | 0.005 |
| Depth+ WS*Below Stream+ Salmon*Middle+ WS*Middle+ Salmon*Lower | 15 | -403.12 | 837.49 | 3.42 | 0.005 |
| Age+ Below Stream+ Depth+ WS*Middle+ WS*Lower | 13 | -405.28 | 837.50 | 3.43 | 0.005 |
| Below Stream+ Depth+ Salmon*Middle+ WS*Middle+ WS*Lower | 14 | -404.20 | 837.50 | 3.43 | 0.005 |
| Below Stream+ Middle+ Depth+ Salmon*Lower+ WS*Lower | 13 | -405.29 | 837.54 | 3.47 | 0.005 |
| Middle+ Lower+ Depth+ WS+ Salmon*Below Stream | 12 | -406.36 | 837.54 | 3.47 | 0.005 |
| Middle+ Lower+ Depth+ Salmon*Below Stream+ WS*Below Stream | 13 | -405.30 | 837.55 | 3.48 | 0.005 |
| Depth+ Salmon+ Mass+ WS*Below Stream+ WS*Lower | 13 | -405.30 | 837.55 | 3.48 | 0.005 |
| Age+ Depth+ WS*Below Stream+ Salmon*Middle+ Salmon*Lower | 15 | -403.15 | 837.57 | 3.50 | 0.005 |
| Age+ Middle+ Lower+ Depth+ Salmon+ WS*Below Stream | 13 | -405.32 | 837.60 | 3.52 | 0.005 |
| Depth+ Salmon*Below Stream+ WS*Lower | 12 | -406.40 | 837.61 | 3.54 | 0.005 |
| Middle+ Lower+ Depth+ Temperature+ Salmon+ WS*Below Stream | 13 | -405.33 | 837.61 | 3.54 | 0.005 |
| Age+ Depth+ Salmon+ WS*Below Stream+ WS*Lower | 13 | -405.34 | 837.62 | 3.55 | 0.005 |
| Depth+ WS*Below Stream+ WS*Middle+ Salmon*Lower+ WS*Lower | 15 | -403.18 | 837.63 | 3.55 | 0.005 |
| Middle+ Lower+ Depth+ Salmon+ Mass+ WS*Below Stream | 13 | -405.34 | 837.63 | 3.56 | 0.005 |
| Depth+ Salmon*Below Stream+ WS*Below Stream+ WS*Lower | 13 | -405.35 | 837.65 | 3.58 | 0.005 |
| Age+ Below Stream+ Depth+ WS+ Salmon*Middle+ Salmon*Lower | 14 | -404.28 | 837.66 | 3.59 | 0.005 |
| Below Stream+ Depth+ Temperature+ WS*Middle+ WS*Lower | 13 | -405.36 | 837.66 | 3.59 | 0.005 |
| Depth+ Temperature+ Salmon+ WS*Below Stream+ WS*Lower | 13 | -405.36 | 837.68 | 3.61 | 0.005 |
| Depth+ Temperature+ WS*Below Stream+ Salmon*Middle+ Salmon*Lower | 15 | -403.24 | 837.74 | 3.67 | 0.005 |
| Middle+ Depth+ WS*Below Stream+ Salmon*Lower+ WS*Lower | 14 | -404.33 | 837.76 | 3.69 | 0.005 |
| Below Stream+ Depth+ Mass+ WS+ Salmon*Middle+ Salmon*Lower | 14 | -404.35 | 837.80 | 3.73 | 0.004 |
| Depth+ Mass+ WS*Below Stream+ Salmon*Middle+ Salmon*Lower | 15 | -403.28 | 837.81 | 3.74 | 0.004 |
| Below Stream+ Middle+ Depth+ Salmon+ Mass+ WS*Lower | 13 | -405.43 | 837.81 | 3.74 | 0.004 |
| Below Stream+ Depth+ Temperature+ WS+ Salmon*Middle+ Salmon*Lower | 14 | -404.37 | 837.84 | 3.77 | 0.004 |
| Below Stream+ Depth+ Salmon*Middle+ WS*Middle+ Salmon*Lower | 14 | -404.38 | 837.86 | 3.79 | 0.004 |
| Age+ Depth+ Salmon+ WS*Below Stream+ WS*Middle+ WS*Lower | 15 | -403.30 | 837.87 | 3.79 | 0.004 |
| Age+ Below Stream+ Middle+ Depth+ Salmon+ WS*Lower | 13 | -405.46 | 837.87 | 3.80 | 0.004 |
| Depth+ Salmon*Below Stream+ WS*Below Stream+ Salmon*Middle+ Salmon*Lower | 15 | -403.31 | 837.88 | 3.81 | 0.004 |
| Below Stream+ Middle+ Depth+ Temperature+ Salmon+ WS*Lower | 13 | -405.48 | 837.91 | 3.84 | 0.004 |
| Depth+ Salmon+ Mass+ WS*Below Stream+ WS*Middle+ WS*Lower | 15 | -403.34 | 837.94 | 3.87 | 0.004 |
| Age+ Below Stream+ Lower+ Depth+ Mass+ WS | 11 | -407.63 | 837.94 | 3.87 | 0.004 |
| Below Stream+ Lower+ Depth+ Temperature+ Mass+ WS | 11 | -407.63 | 837.94 | 3.87 | 0.004 |
| Depth+ WS+ Salmon*Below Stream+ Salmon*Middle+ Salmon*Lower | 14 | -404.44 | 837.98 | 3.91 | 0.004 |
| Middle+ Depth+ Salmon*Below Stream+ WS*Lower | 13 | -405.53 | 838.00 | 3.93 | 0.004 |
| Depth+ Salmon*Below Stream+ WS*Below Stream+ WS*Middle+ WS*Lower | 15 | -403.37 | 838.00 | 3.93 | 0.004 |
| Below Stream+ Depth+ Salmon*Middle+ WS*Middle+ Salmon*Lower+ WS*Lower | 15 | -403.37 | 838.01 | 3.94 | 0.004 |
| Depth+ Temperature+ Salmon+ WS*Below Stream+ WS*Middle+ WS*Lower | 15 | -403.38 | 838.03 | 3.96 | 0.004 |
| Below Stream+ Depth+ Mass+ WS+ Salmon*Lower | 12 | -406.63 | 838.07 | 4.00 | 0.004 |

Table headings described in Table S3.
