# Supplementary figures and images for: Location Is Everything: Evaluating the Effects of Terrestrial and Marine Resource Subsidies on an Estuarine Bivalve
Source: PLoS One. 2015 May 18;10(5):e0125167. doi: 10.1371/journal.pone.0125167 (PMC4436346; doi:10.1371/journal.pone.0125167)

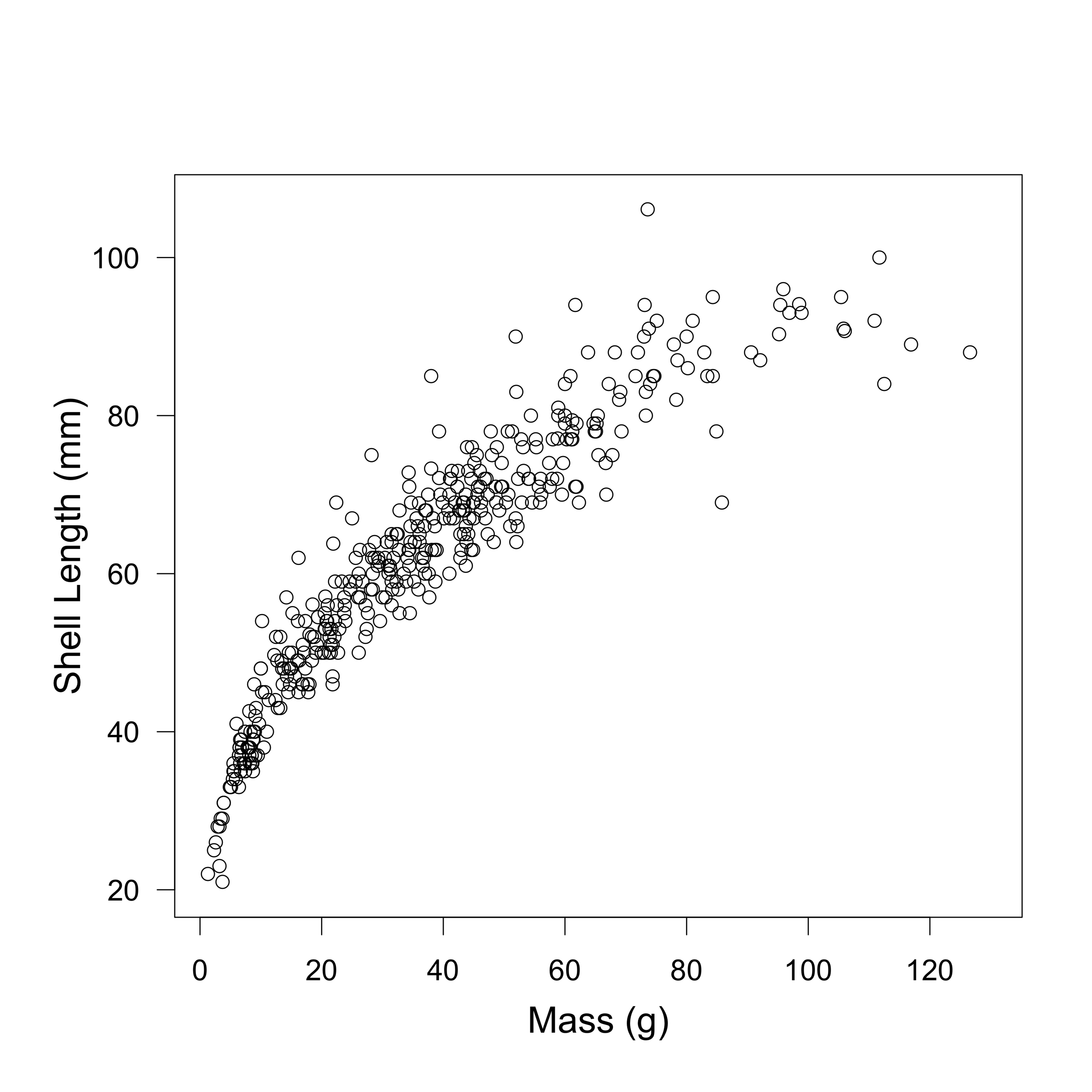

Supplement: S1 Fig — (TIF) [file pone.0125167.s010.tif]
